# Sustainable MnO_2_/MgO Bimetallic Nanoparticles Capped with Sword Fern Methanol Extract Attain Antioxidant/Anti-Biofilm Potential: A UPLC-ESI/LC/MS and Network Pharmacology-Supported Study

**DOI:** 10.3390/ph18091262

**Published:** 2025-08-25

**Authors:** Esraa A. Elhawary, Raya Soltane, Mohamed H. Moustafa, Amer Morsy Abdelaziz, Mohamed A. Said, Eman Maher Zahran

**Affiliations:** 1 Department of Pharmacognosy, Faculty of Pharmacy, Ain Shams University, Cairo 11566, Egypt; esraa.elhawary@pharma.asu.edu.eg; 2 Department of Biology, Adham University College, Umm Al-Qura University, Makkah 21955, Saudi Arabia; 3 College of Pharmacy, Al-Farahidi University, Baghdad 00964, Iraq; mohamed.hamed@uoalfarahidi.edu.iq; 4 Botany and Microbiology Department, Faculty of Science, Al-Azhar University, Cairo 11884, Egypt; amermorsy@azhar.edu.eg; 5 Department of Pharmaceutical Chemistry, Faculty of Pharmacy, Egyptian Russian University, Cairo 11829, Egypt; mohamed-adel@eru.edu.eg; 6 College of Pharmacy, University of Kut, Wasit 52001, Iraq; 7 Department of Pharmacognosy, Faculty of Pharmacy, Deraya University, Minia 61111, Egypt

**Keywords:** eco-friendly, *Nephrolepis exaltata*, UPLC-ESI/LC/MS, bimetallic nanoparticles, anti-biofilm, network pharmacology

## Abstract

**Background**: *Nephrolepis exaltata* (sword fern) possesses a considerable amount of phytochemicals and different biological activities. The current study investigates the anti-biofilm potential of greenly synthesized bimetallic nanoparticles of *Nephrolepis exaltata* leaf methanol extract (NEME-MnO_2_-MgO BNPs). **Methods**: The NEME was subjected to UPLC/MS analysis, followed by characterization of its NPs by size, zeta potential, FTIR, entrapment efficiency, and release. Then, antioxidant, antimicrobial and antibiofilm assays were employed, followed by in silico studies. **Results**: The UPLC/MS analysis of NEME led to the tentative identification of 27 metabolites, mostly phenolics. The MnO_2_-MgO BNPs presented a uniform size and distribution and exhibited IC_50_ values of 350 and 215.6 μg/mL, in the DPPH and ABTS assays, respectively. Moreover, the NPs exhibited antimicrobial and anti-biofilm efficacies against *Pseudomonas aeruginosa*, *Klebsiella pneumonia* (ATCC-9633), *Staphylococcus aureus* (ATCC-6538), *Escherichia coli*, *Bacillus cereus*, and *C. albicans*, with MIC values of 250–500 μg/mL. The MnO_2_-MgO BNPs inhibited *Candida albicans* biofilms with a % inhibition of 66.83 ± 2.45% at 1/2 MIC. The network pharmacology highlighted epigallocatechin and hyperoside to be the major compounds responsible for the anti-biofilm potential. The ASKCOS facilitated the prediction of the redox transformations that occurred in the green synthesis, while the docking analysis revealed enhanced binding affinities of the oxidized forms of both compounds towards the outer membrane porin OprD of *P. aeruginosa*, with binding scores of −4.6547 and −5.7701 kcal/mol., respectively. **Conclusions**: The greenly synthesized *Nephrolepis exaltata* bimetallic nanoparticles may provide a promising, eco-friendly, and sustainable source for antimicrobial agents of natural origin with potential biofilm inhibition.

## 1. Introduction

Ferns are crucial to the preservation of moisture in forests because their roots absorb water and progressively disperse it throughout the soil and atmosphere. This encourages the growth of microflora and substrate microfauna, both of which are critical to maintaining the ecological balance of the ecosystem. They directly impact the microclimate in many places since they have been used as ornaments, tools for religious rites and entertainment, tools for maintaining the fauna in various locations, and food, as well as refuge for a variety of species [1]. In addition to their aesthetic appeal, ferns are crucial for nutrition and economy. Because they endanger biodiversity, certain fern species are viewed as intruders and handled like diseases. Some species, mostly arborescent ones, are excellent examples of how ferns interact with other organisms because their rachises may be surrounded by a variety of other plant species, including bryophytes, pteridophytes, and orchidaceous ones, as well as small animals like ants and microscopic and macroscopic fungi [1].

Thirty taxa of ferns make up the genus *Nephrolepis* (Nephrolepidaceae), which is extensively dispersed, primarily in tropical regions of Asia, Africa, and South America. One of the common decorative fern species, *Nephrolepis exaltata* (L.) Schott, grows well in cool and humid environments and reproduces quickly. This plant is sold as “sword fern, Boston Fern, or Bostoniensis” and is mostly grown for horticulture [2]. *Nephrolepis exaltata* is the least researched of the 30 species in the genus *Nephrolepis* that are therapeutically beneficial. The division Tracheophyta includes the genus *Nephrolepis* (Nephros, kidney; lepis, the indusium kidney-shaped and scale-like), which is further divided into the subdivision Polypodiophytina, class Polypodiopsida, and order Polypodiales.

*Nephrolepis exaltata*, commonly known as the sword fern, is an ornamental plant in most of India and the world. *N. exaltata* contains alkaloids, flavonoids, carbohydrates, saponins, phenols, and sterols [3]. The volatile components identified from *N. exaltata* have exhibited potent antimicrobial activities against a number of selected microorganisms, with some promising cytotoxicity potential on lung, breast, and colon carcinoma cells [4].

Biofilms, established by some microorganisms, are complex communities that are capped with exopolysaccharide (EPS) layers [5]. These bacterial communities can interact with one another and stick to surfaces, becoming stronger and more effective in navigating the harsh environment in which they develop [6]. It is important to remember that once a biofilm forms, the bacteria are shielded from immune defense mechanisms and physical or chemical therapeutic strategies and become more violently resistant. As more resistant microbial strains have emerged during the past 20 years, numerous determinants of resistance have been identified [7].

Nanotechnology has gained global attention due to the unique characteristics of nanoparticles (NPs), including variant shapes, small sizes, and high surface areas, making NPs suitable for a wide range of medical applications [8]. Conventional synthesizing approaches for NPs are generally time-consuming, expensive, need specific precursors and definite temperatures, and create significant chemical waste [9]. In contrast, green synthesis utilizes renewable organic extracts which eliminate the need for toxic chemicals and gives rise to waste production. By employing natural products rich with secondary metabolites, the green approach provides a sustainable alternative for NP production, aligning with recent sustainability concerns [10].

To the best of our knowledge, *Nephrolepis exaltata* has been rarely investigated in combination with nanotechnology. Greenly synthesized iron oxide NPs of *N. exaltata* have been previously investigated for antiplasmodial and cytotoxic effects [11]. Additionally, greenly synthesized zinc oxide NPs of *N. exaltata* have been previously studied for antiplatelet and cytotoxic potential [12]. Hence, the current study is considered unique and comprehensive, employing green approaches in manufacturing bimetallic NPs of *N. exaltata*, offering an eco-friendly, sustainable, and highly potent formulation attaining antioxidant and antimicrobial potential.

**Figure 1:**
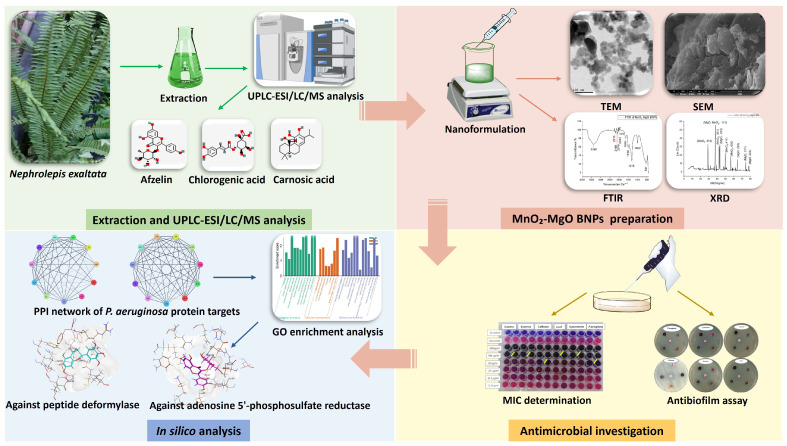
Graphical representation of the phytochemical and antimicrobial studies on *Nephrolepis exaltata*.

Collectively, our study aims to define the phytochemical profile of the methanol extract of *Nephrolepis exaltata* leaves via UPLC/MS techniques, as well as evaluate its antioxidant, antimicrobial, and biofilm potential using greenly synthesized MnO_2_-MgO biometallic nanoparticles (Figure 1).

## 2. Results and Discussion

### 2.1. UPLC/MS Analysis for the Methanol Leaves Extract of Nephrolepis exaltata

**Figure 2:**
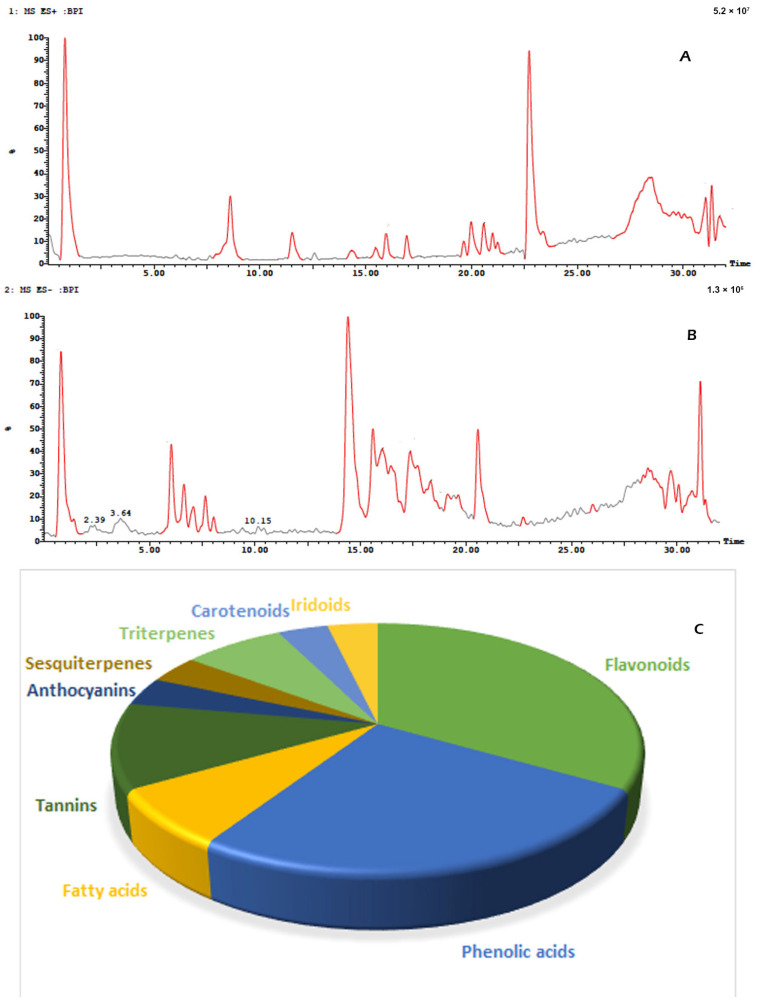
(**A**) UPLC-ESI/LC/MS positive mode chromatogram. (**B**) UPLC-ESI/LC/MS negative mode chromatogram of *Nephrolepis exaltata* methanol extract. (**C**) Pie chart showing the % of components identified via UPLC-ESI/LC/MS analysis.

**Figure 3:**
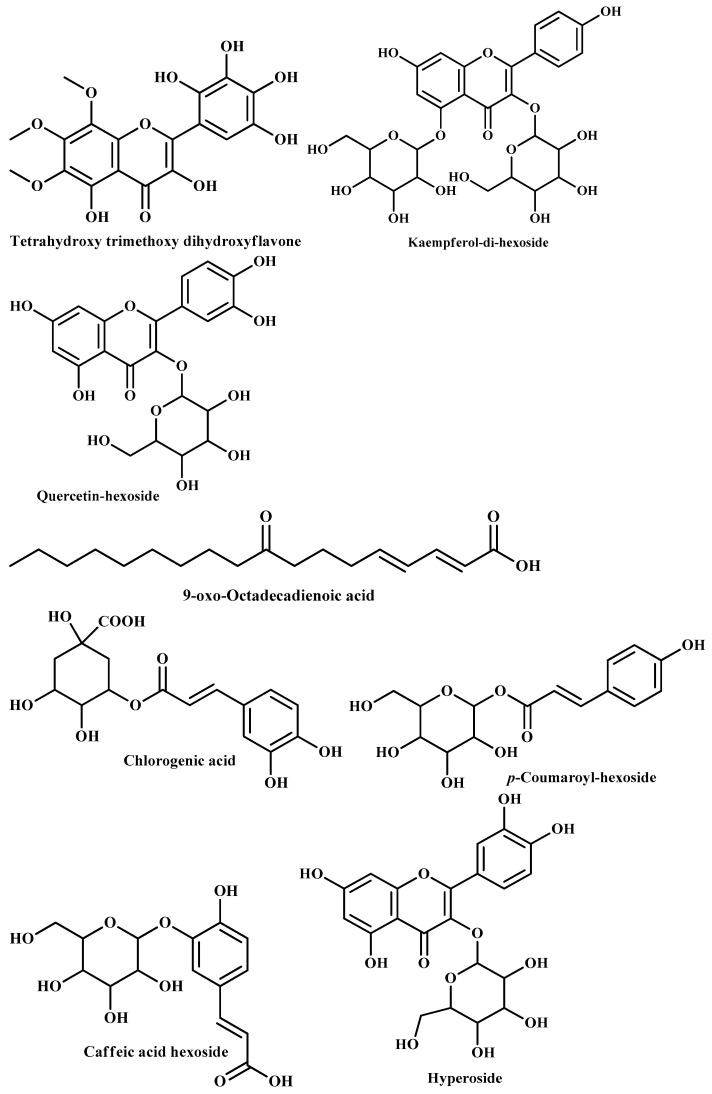
Structures of some metabolites identified by UPLC-ESI/LC/MS.

Twenty-seven metabolites were detected and tentatively identified from the methanol extract of *N. exaltata* leaves, expressed in both positive and negative modes (Figure 2A,B). The tentatively identified metabolites are listed in Table 1 according to their retention time, and their *m*/*z* values, together with molecular formula, chemical class, and area %, are also included. The most abundant classes were the flavonoids, phenolic acids, tannins, and triterpenes, in addition to other phytoconstituents, *viz*. anthocyanins, iridoids, and fatty acids (Figure 2C). The identification was based on comparison of the *m*/*z* values with the relevant literature. The identified classes of phytoconstituents are summarized in Figure 3.

Flavonoids represented the most predominant class of identified components, with nine tentatively identified compounds, where kaempferol and myricetin were the common aglycones. Compound 1 presented a deprotonated peak at *m*/*z* 377 (12.03%) and at *m*/*z* 381 (18.13%), and it appeared to be the flavone named tetrahydroxy trimethoxy dihydroxyflavone [13,14]. Compounds 2, 3, and 6 were detected at *m*/*z* 609, 447, and 459, respectively, and they were tentatively defined as kaempferol-*di*-hexoside [15], kaempferol-hexoside [15], and kaempferol-hexuronoide [15] (Table 1). Similarly, myricetin aglycone (*m*/*z* 319, +ve mode) [16] and its glycoside, myricetin-pentosyl pentoside [*m*/*z* 609(611)], [16] were tentatively identified. A famous flavonoid, previously reported from genus *Nephrolepis*, appeared in the ESI negative ion mode at *m*/*z* 431 and was then defined as afzelin [2]. In addition to that, one major peak was detected at *m*/*z* 463 with an area % of 8.15% which was tentatively defined as quercetin-hexoside [15]. A deprotonated molecular ion peak was detected at [M − H]^−^ *m*/*z* 459 and at [M + H]^+^ *m*/*z* 461 and it was tentatively assigned to glycitein-hexouronide [24,25].

Phenolic acids represented the second most abundant class of phytoconstituents, where seven components were tentatively identified. One major peak was detected at *m*/*z* 353 in ESI negative mode (5.49%) and at *m*/*z* 355 in ESI positive mode (6.40%) which was defined as caffeoyl quinic acid or chlorogenic acid [13,14,19,31]. Further, a chlorogenic acid dimer presented a deprotonated peak at *m*/*z* 707 (2.41%), which was defined as caffeoyl quinic acid dimer [30,32]. Other caffeic acid derivatives were also traced, *viz*. caffeic acid hexoside at [M − H]^−^ *m*/*z* 341 (6.22%) and at [M + H]^+^ *m*/*z* 347 (2.75%) [14,20]. Moreover, a deprotonated peak was traced at *m*/*z* 591 in ESI negative ion mode which was tentatively assigned to benzoyl caffeic acid rutinoside [18]. Compound 15 was detected at *m*/*z* 325 (11.43%) and was identified as *p*-coumaroyl-hexoside [16,33]. Two simple phenolic acids presented their peaks at *m*/*z* 333 (positive mode) and at *m*/*z* 313 (315), which were tentatively named as carnosic acid [14,21] and methoxy benzoic acid (*p*-anisic acid) [16], respectively.

Two fatty acid peaks were defined from the plant extract. An abundant peak (20.80%) appeared at *m*/*z* 293 in ESI negative mode which was identified as 9-*oxo*-octadecadienoic acid [17,18]. Moreover, a deprotonated peak was detected at [M + H]^+^ *m*/*z* 285 and it belonged to the fatty acid ester, ethyl palmitate (2.04%) [16].

Interestingly, three tannins were tentatively identified, where one tannin peak was traced at *m*/*z* 637 (+ve mode), which was defined as trigalloyl hexoside [22]. Compound 28 presented a deprotonated peak at *m*/*z* 481 (1.10%), which was identified as galloyl-HHDP [27]. In addition, compound 34 had a deprotonated molecular ion peak at [M − H]^−^ *m*/*z* 305 and [M + H]^+^ *m*/*z* 307, which lead to its identification as epigallocatechin, which was also documented before from the genus *Nephrolepis*. Oleanolic acid, the famous triterpene which was reported from the genus *Nephrolepis*, showed a deprotonated peak at *m*/*z* 457, C_30_H_48_O_3_ [29]. Further, another peak was traced at *m*/*z* 487 for methoxy ursolic acid (Table 1) [28].

In our current study, the NEME was phytochemically analyzed through UPLC/MS, which lead to the tentative identification and quantification of 27 metabolites, most of which are flavonoids, phenolic acids, tannins, triterpenes, and other classes. Upon reviewing literature on genus *Nephrolepis*, trace reports were detected regarding the phytochemical analysis and isolation of phytoconstituents, and they were summarized as follows. Different phytoconstituents were isolated from the leaves of *Nephrolepis exaltata*, including phloretic acid, dehydrovomifoliol, dehydrololiolide, quercitrin, kaempferol, afzelin, quercetin, luteolin, 3-*O*-methylquercetin, demethoxymatteucinol, methoxygaertneroside, (2S)-5,7,3′,5′-tetrahydroxyflavanone, epigallocatechin, lanicepside A, pinoresinol 4′-*O*-*β*-D-glucopyranoside, and glochidiobioside [2]. Moreover, zinc oxide nanoparticles (ZnO-NPs) synthesized from an aqueous extract of *Nephrolepis exaltata* demonstrated potential antiplatelet activity by inhibiting the platelet aggregation induced by platelet activation factor (PAF) and arachidonic acid (AA). The results showed that synthesized ZnO-NPs were more effective in inhibiting platelet aggregation induced by AA with IC50 (56% and 10 μg/mL) and PAF (63% and 10 μg/mL), respectively. The cytotoxicity of synthesized nanoparticles revealed that cell viability decreased and the IC50 was found to be 46.7% at a concentration of 75 μg/mL [12]. In another study, the aerial parts of *Nephrolepis biserrata* and *Nephrolepis cordifolia* were fractionated in different solvents. These fractions were concentrated to obtain a powder and were tested against nine bacterial and three fungal strains according to the disc diffusion method. The water and methanol fractions were active against most of the tested bacterial and fungal strains, and some of these were more effective than the controls tested [34].

The fresh leaves of NEME revealed the presence of alkaloids, flavonoids, tannins, saponins, phlobatanins, steroids, anthraquinone, and cyanogenic gylcosides. The proximate composition showed a considerable amount of crude fiber, crude protein, ash content, carbohydrate, and energy content. The results of the mineral element content revealed that the fern contained a high quantity of potassium, phosphorus, calcium, magnesium, and iron and a moderate amount of manganese and zinc. Anti-nutrient analysis showed low concentrations of cyanide (0.06 ± 0.01 mg/100 g), phytate (0.25 ± 0.01 mg/100 g), and oxalate (0.69 ± 0.01 g/100 g) [35].

### 2.2. MnO_2_-MgO BNPs Biosynthesis Using NEME

In the current study, NEME was utilized to create MnO_2_-MgO BNPs from Mn acetate tetra hydrate and Mg nitrate hexahydrate, all stabilized in a colloidal condition, with the solution’s color changed to black [36]. The phytochemical composition of NEME plays a critical role in the biosynthesis of the NPs, due to presence of active constituents which contribute to metal ion reduction. This process occurs through tautomeric shifts, where enol forms transition into keto forms, releasing reactive hydrogen atoms that aid in nanoparticle formation [11,37]. This is consistent with a previous study which reported the successful formulation of zinc oxide nanoparticles using the aqueous extract of *Nephrolepis exaltata* as both a reducing and capping agent, via using zinc acetate dihydrate (Zn(CH_3_CO_2_)_2_·2H_2_O) as the zinc precursor [12]. Additionally, The iron oxide nanoparticles (FeO NPs) from NEME were also formed and showed potent antiplasmodial activity with an MIC of 62 ± 1.3 at 25 µg/mL against Plasmodium parasites [11].

### 2.3. Characterization of the Biosynthesized MnO_2_-MgO BNPs

**Figure 4:**
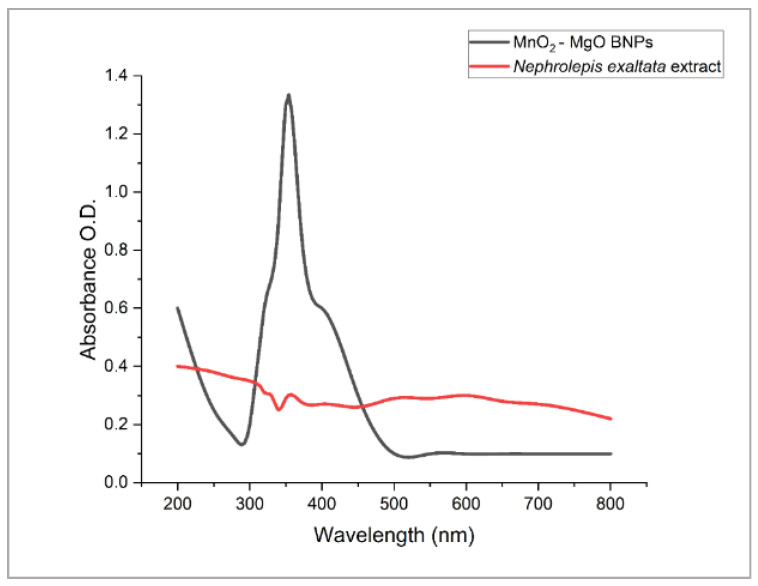
UV-Vis spectroscopy of MnO_2_-MgO BNPs and NEME.

The potential of NEME to biosynthesize MnO_2_-MgO BNPs was visually assessed via the color change from yellow to black. This color change indicated the activation of SPR in the biogenic MnO_2_-MgO BNPs, producing a black color that was recognized as the characteristic spectroscopic signature for their successful formation. Based on UV-Vis investigations, the experimental peak conducting the OD (1.3; diluted twice) of the MnO_2_-MgO BNPs was observed at 350 nm (Figure 4). On the other hand, the peak of the NEE disappeared from the UV-Vis spectrum, which further confirms the optimal BNP formation.

Generally, the structure, dielectric characteristics, morphological surfaces, intensity, and size of any generated nanoparticles are frequently critical factors that greatly affect the SPR. The obtained results were matched with the reported ones about the absorption peaks of MnO_2_ nanoparticles, which generally appear at 350–400 nm, corresponding to charge transfer transitions between Mn^3+^ and Mn^4+^ states [38]. Alwin David and Ram Kumar [39] reported that the UV–visible spectrum of biosynthesized MnO_2_ nanoparticles exhibited an absorption maximum at 371 nm, confirming the characteristic surface plasmon resonance of MnO_2_ NPs. Farhan and Mohammed [40] reported that the UV–visible spectrum of MnO_2_ nanoparticles exhibited a broad absorption band with two characteristic peaks at approximately 320 nm, indicating the successful formation of MnO_2_ NPs.

Additionally, Faisal et al. [41] investigated the green synthesis of MnO_2_ nanoparticles using *Fagonia cretica* and reported a distinct absorption peak at 410 nm with an absorbance of 2.25 a.u., confirming the successful formation of the nanoparticles.

Similarly, MgO nanoparticles exhibit a strong absorption band appeared in the 200–300 nm range, which is usually associated with excitonic transitions and oxygen vacancies [42]. Hassan et al. [43] reported that magnesium oxide nanoparticles synthesized via *Rhizopus oryzae*-mediated green synthesis exhibited a surface plasmon resonance (SPR) peak at 282 nm, confirming the formation of MgO NPs. Abdallah et al. [44] and Nguyen et al. [45] reported that the maximum absorption peaks of MgO nanoparticles synthesized using *Rosmarinus officinalis* L. and *Tecoma stans* (L.) appeared at 250 nm and 281 nm, respectively. The presence of both characteristic peaks in the UV–visible spectra confirms the formation of MnO_2_-MgO bimetallic nanoparticles. Additionally, the peak shifts appearing as broadening in the UV spectrum indicate NP interactions and stabilization by plant bioactive constituents during the synthesis [46].

**Figure 5:**
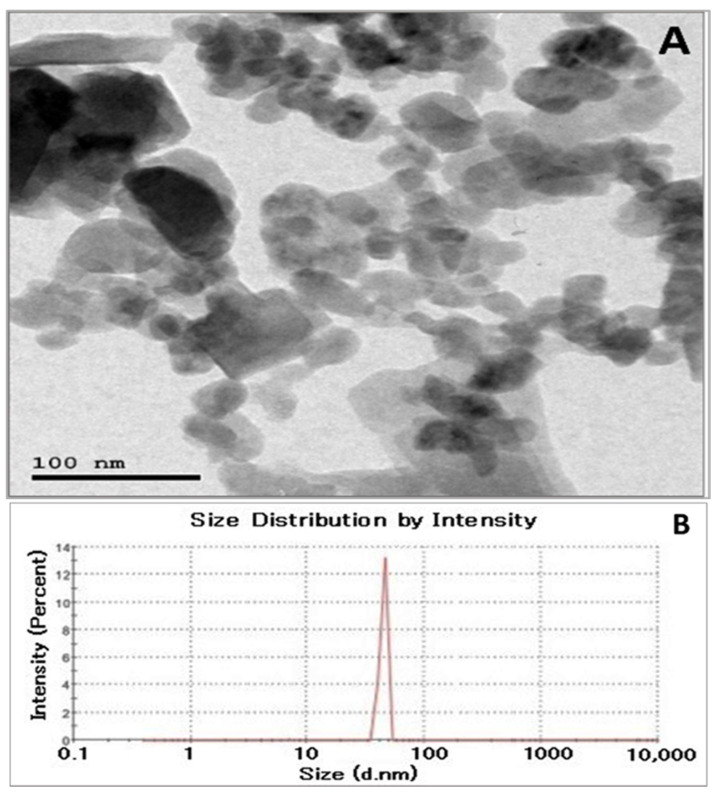
Investigation of (**A**) TEM and (**B**) DLS images of MnO_2_-MgO BNPs.

The TEM image presents the morphology of the MnO_2_-MgO BNPs, where the nanoparticles exhibited a heterogeneous distribution with varying shapes, including quasi-spherical and irregular shapes, with the presence of a notable agglomeration, likely due to interparticle interactions. The scale bar of 100 nm confirms the nanoscale nature of the synthesized material, with sizes varying between 20 and 80 nm, as depicted in the TEM image (Figure 5A). These variations in shape are often attributed to differences in nucleation and growth kinetics during the synthesis, which are affected by the plant extract’s bioactive constituents and reaction conditions. The presence of agglomeration suggests that while the plant-derived reducing and stabilizing constituents helped in the NP formation, their capping force might not have been sufficient to completely prevent interparticle interactions [47]. In the MnO_2_-MgO BNPs biosynthesized by the NEE, the particle size distribution characterized by the DLS approach was found to be between 50 and 90 nm, with an average size of 70 nm as determined on a log-scale intensity plot (Figure 5B). Differences between TEM and DLS are frequently reported due to their respective measurement principles, where DLS reflects hydrodynamic diameter and is often larger than the physical size observed by TEM [48]. Since PDI values exceeding 0.7 express polydispersity particle diffusion, our results showed a PDI value of 0.571 [49]. Collectively, the results confirm that the biosynthesized MnO_2_-MgO BNPs exhibit a uniform and homogeneous size distribution.

**Figure 6:**
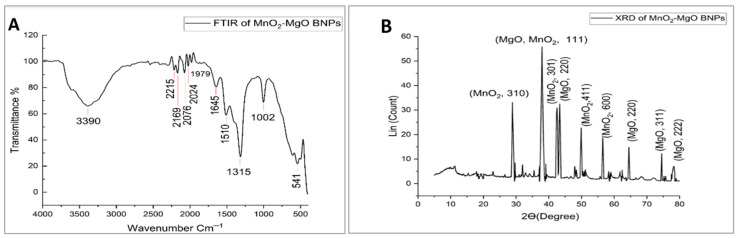
(**A**) FTIR of MnO_2_-MgO BNPs synthesized from *Nephrolepis exaltata* extract; and (**B**) XRD of MnO_2_-MgO BNPs.

The FTIR spectrum of MnO_2_-MgO BNPs of NEME (Figure 6A) reveals distinct absorption bands which confirm the presence of functional groups derived from the extract, all of which play a crucial role in the metal oxide formation. The broad absorption band is attributed to O-H and N-H stretching vibrations, signifying the presence of OH and NH from the plant bioactive constituents. These functional groups suggest the involvement of flavonoids, tannins, and proteins in reducing and stabilizing MnO_2_-MgO BNPs [50]. The FTIR results and previous studies highlight the effectiveness of plant-mediated (green) synthesis in producing stable and functional metal oxide NPs with potential applications in agriculture and biomedicine [51,52].

The XRD diffractograms of the synthesized MnO_2_-MgO BNPs confirm their crystalline nature through distinct diffraction peaks corresponding to various lattice planes (Figure 6B). The observed diffraction peaks at 2θ values of 28.9°, 37.9°, 42.5°, 43.3°, 49.9°, 56.5°, 64.99°, 74.5°, and 78.3° are indexed to the respective lattice planes (310), (111), (301), (220), (411), (600), (220), (311), and (222). These reflections are aligned with the standard crystallographic data for MgO and MnO_2_, indicating the successful formation of the BNPs. The peaks at 37.9°, 43.3°, 64.99°, 74.5°, and 78.3° correspond to the Bragg’s reflection planes (111), (220), (220), (311), and (222), which are characteristic for the FCC lattice structure of MgO, suggesting a well-defined crystalline phase [53]. The combination of structural confirmation through XRD and the FTIR results, reinforces the potential of MnO_2_-MgO BNPs as functional nanomaterials characterized by enhanced stability and reactivity. The diffraction peaks at 28.9° and 42.5° can be specifically attributed to MnO_2_, which aligns with the tetragonal and orthorhombic phases of manganese dioxide, confirming its presence within the synthesized BNPs [54].

**Figure 7:**
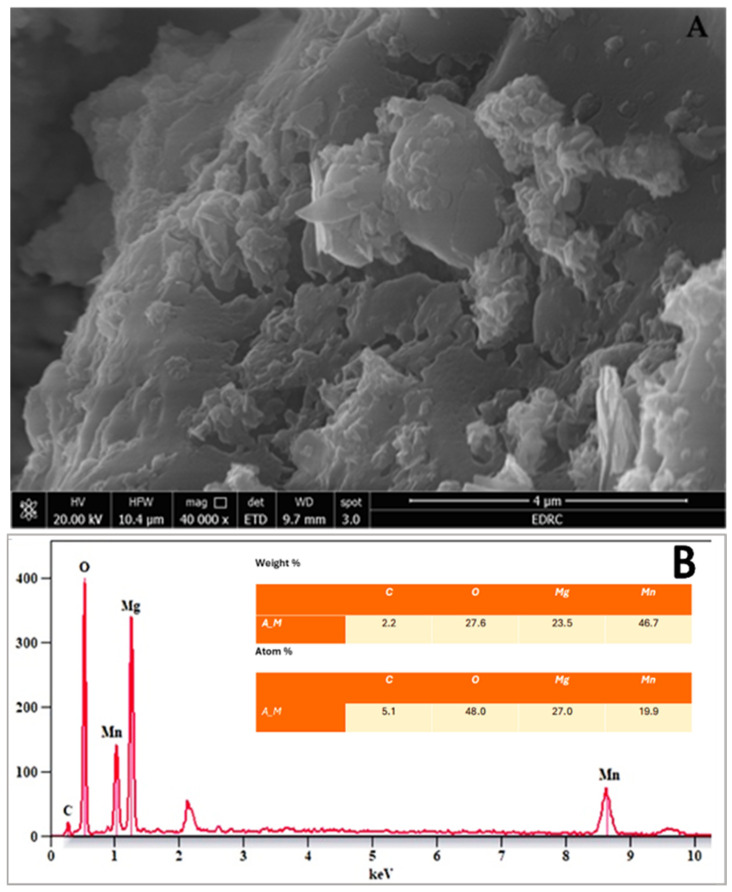
(**A**) SEM micrograph (scale bar = 4 µm, magnification = 40,000×) and (**B**) EDX spectrum showing the elemental composition of MnO–MgO BNPs biosynthesized using *Nephrolepis exaltata* extract.

The SEM image (Figure 7A) of the MnO_2_–MgO BNPs shows a heterogeneous morphology characterized by agglomerated structures. The BNPs appear as irregularly shaped clusters, with certain areas exhibiting well-defined, plate-like crystalline formations. These aggregated structures are likely the result of nanoparticle clustering during the drying and preparation process for SEM analysis. On the other hand, the EDX analysis (Figure 7B) confirms the elemental composition of the NEE-synthesized nanostructures. The spectrum displays characteristic peaks corresponding to Mn, Mg, O, and C, which reveals the successful formation of MnO_2_-MgO BNPs. The weight % analysis shows that Mn (46.7 wt. %) and Mg (23.5 wt. %) are the predominant elements. The observed morphology aligns with the reported data on the biosynthesized metal oxide NPs, where plant extracts influence NPs’ shape and stabilization. The presence of both aggregated and structured NPs indicates that while the synthesis approach effectively produces BNPs, further optimization such as surfactant assisted synthesis and/or ultrasonication could enhance dispersion and diminish particle agglomeration [55].

### 2.4. The In Vitro Antimicrobial Assay

**Figure 8:**
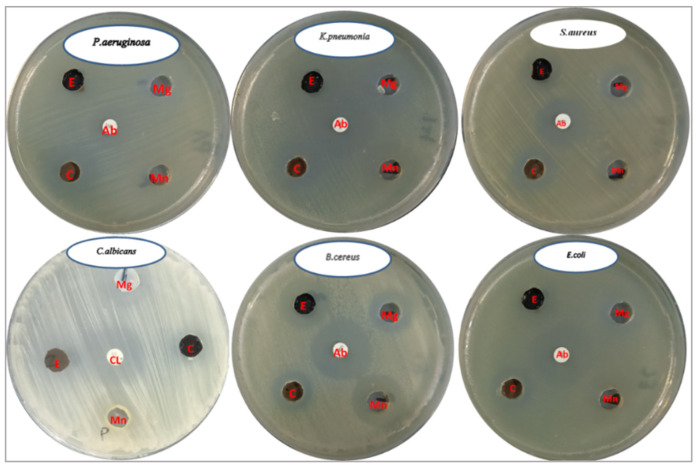
Antimicrobial activity of E, NEME; Mg, Mg nanoparticles; Mn, Mn nanoparticles; and C, MnO_2_-MgO BNPs against *Pseudomonas aeruginosa*, *Klebsiella pneumonia* (ATCC-9633), *Staphylococcus aureus* (ATCC-6538), *Escherichia coli*, *Bacillus cereus*, and *Candida albicans* (ATCC-10231).

Table 2 summarizes the diameter of inhibition zones (in mm) for various microbial strains when exposed to *Nephrolepis exaltata* extract, magnesium nanoparticles, manganese nanoparticles, and MnO_2_-MgO BNPs, with the results compared to standard antimicrobials (chloramphenicol/clotrimazole). This agar well diffusion assay, a widely accepted method, measures antimicrobial potency based on the size of the clear zone where microbial growth is inhibited around the tested substance. The MnO_2_-MgO BNPs were active against both Gram-positive and Gram-negative bacteria, as well as against *C. albicans* with different inhibition zone diameters, as mentioned in Table 2 and Figure 8.

### 2.5. Determination of MIC

**Figure 9:**
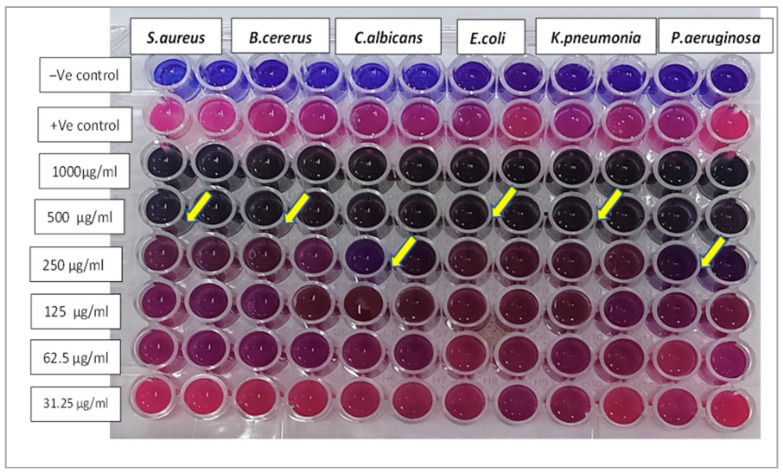
MIC of MnO_2_-MgO BNPs against *Pseudomonas aeruginosa*, *Klebsiella pneumoniae* (ATCC-9633), *Staphylococcus aureus* (ATCC-6538), *Escherichia coli*, *Bacillus cereus*, and *Candida albicans* (ATCC-10231). The yellow arrows refer to the MIC values of the lowest concentrations required for microbial growth inhibition.

Table 3 shows the MIC values of MnO_2_-MgO BNPs against the tested strains. The MIC values indicate that the lowest concentration required of the MnO_2_-MgO BNPs to inhibit the microbial growth ranged between 250 to 500 μg/mL, as shown in Table 3 and Figure 9.

### 2.6. The Antioxidant Activity

**Figure 10:**
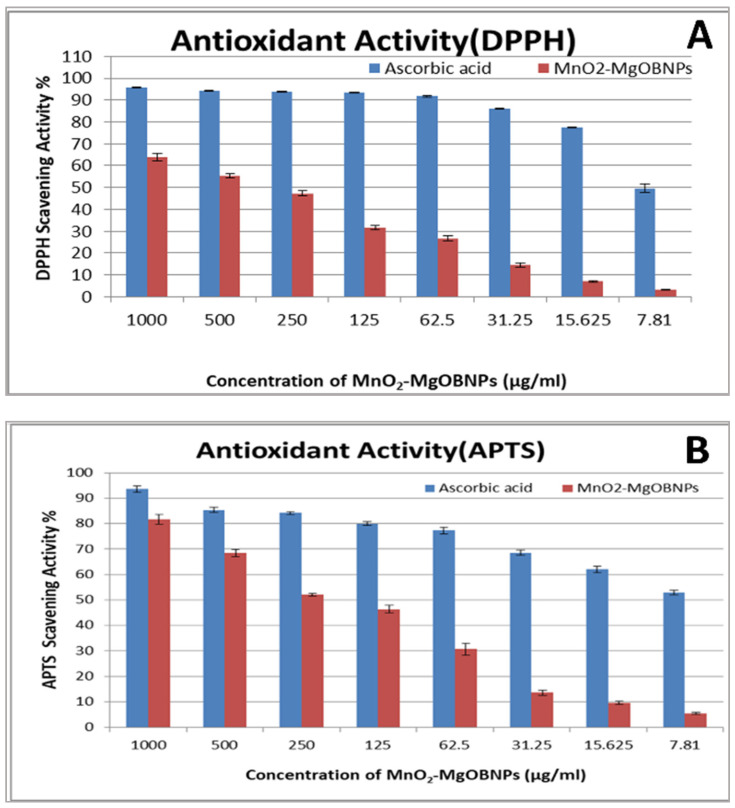
Antioxidant activity of MnO_2_-MgO BNPs at different concentrations, measured using (**A**) DPPH and (**B**) ABTS methods.

In the current study, the antioxidant activity of MnO_2_-MgO BNPs at different concentrations (1000–7.81 μg/mL) was evaluated using the DPPH and ABTS assays, as illustrated in Figure 10A,B, respectively. The results demonstrated that MnO_2_-MgO BNPs exhibited an IC_50_ value of 350 μg/mL in the DPPH assay, while in the ABTS assay they gave an IC_50_ value of 215.6 μg/mL. These findings highlight the potent antioxidant efficacy of MnO_2_-MgO BNPs, which reveals their power to reduce oxidative stress related to excessive ROS generation. It is known that the antioxidants play a crucial role in neutralizing ROS, which are by-products of biological reactions implicated in numerous pathological conditions, including inflammation, cancer, and neurodegenerative diseases [56].

### 2.7. The Anti-Biofilm Activity

**Figure 11:**
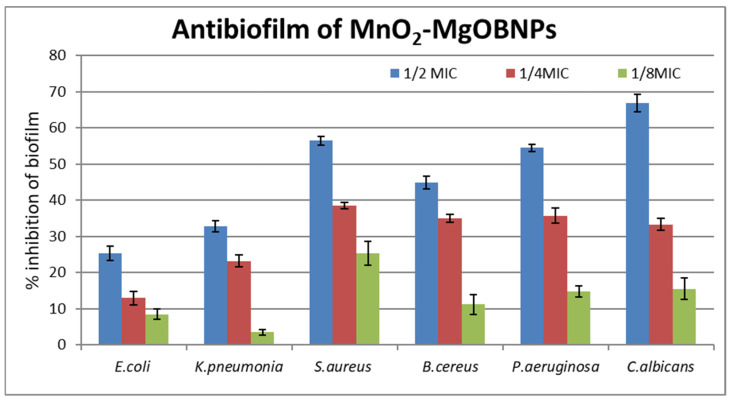
Anti-biofilm activity of MnO_2_-MgO BNPs against tested microorganisms.

Biofilm formation is a crucial survival strategy for both fungi and bacteria that enables them to survive in diverse environments, resist harsh conditions, and escape host immune responses [57]. The in vitro anti-biofilm activity of MnO_2_-MgO BNPs against all the tested pathogens (Figure 11) revealed a concentration-dependent reduction in their biofilm formation. *Candida albicans* exhibited the highest biofilm inhibition, with a reduction ranging from 66.83 ± 2.45% at 1/2 MIC to 15.53 ± 3.03% at 1/8 MIC. In contrast, *Escherichia coli* showed the lowest biofilm inhibition, with percentages ranging from 25.35 ± 2.04% at 1/2 MIC to 8.51 ± 1.41% at 1/8 MIC. The ability of MnO_2_-MgO BNPs to impair the integrity of biofilms suggests their potential use in medical applications, such as in coating medical devices or resisting biofilm-related infections [58]. The significant reduction in the formation of *Candida albicans* biofilm indicates that fungal biofilms may be more susceptible to the action of MnO_2_-MgO BNPs compared to bacterial biofilms. This could be attributed to structural differences between fungal and bacterial biofilms, as the formers often rely on an extensive extracellular matrix that may be more susceptible to nanoparticle-mediated disruption [59].

### 2.8. In Silico Biological Activity Predictions

The integration of in silico approaches with experimental methodologies has significantly advanced the understanding of complex chemical and biological processes. This synergistic strategy has proven particularly effective in identifying molecular targets and elucidating the mechanisms of action of bioactive compounds [60,61]. In the present study, computational techniques were employed to analyze the major constituents of *Nephrolepis exaltata* extract, aiming to elucidate the components responsible for its observed antimicrobial activity against *Pseudomonas* species, as confirmed by biological assay data. Furthermore, predictive modeling was conducted to explore the potential mechanisms underlying this activity. The identified bioactive compounds underwent statistical screening for antimicrobial potential and were further evaluated using the PASS Online platform to predict their antibacterial efficacy, particularly against *Pseudomonas* spp., as detailed in Supplementary Table S2. The results demonstrated a high probability of antibacterial activity (Pa > 0.5) for the majority of the tested compounds. However, the compounds with CIDs 10155076, 157010309, and 90477731 (caffeoylquinic acid, *p*-Coumaroyl-hexoside, and methoxy ursolic acid, respectively) were predicted to lack antibacterial potential (Pa < 0.5). Notably, the compounds with CIDs 72277 (epigallocatechin) and 5281643 (hyperoside) exhibited promising antibacterial activity against *Pseudomonas* sp., with Pa values of 0.7 and 0.5, respectively.

### 2.9. Prediction of the Potential Targets

#### 2.9.1. Prediction of the Potential *E. coli* Targets of the Annotated Compounds

Given the importance of identifying therapeutic targets through which phytochemicals exert antimicrobial activity, the PharmMapper server was utilized to predict potential protein targets using a pharmacophore mapping approach. PharmMapper was employed to identify targets for the compounds with PubChem CIDs 72277 and 5281643. Each compound yielded 298 predicted protein targets (Supplementary Materials, Tables S3 and S4), among which 80 targets were identified as bacterial proteins for CID 72277 and 77 for CID 5281643 (Supplementary Materials, Tables S5 and S6). Upon further analysis using the STRING database, only two protein targets related to *P. aeruginosa* were identified for CID 72277, while one *P. aeruginosa*-associated target was found for CID 5281643 (Table 4).

#### 2.9.2. Protein–Protein Interaction (PPI) Network Analysis

**Figure 12:**
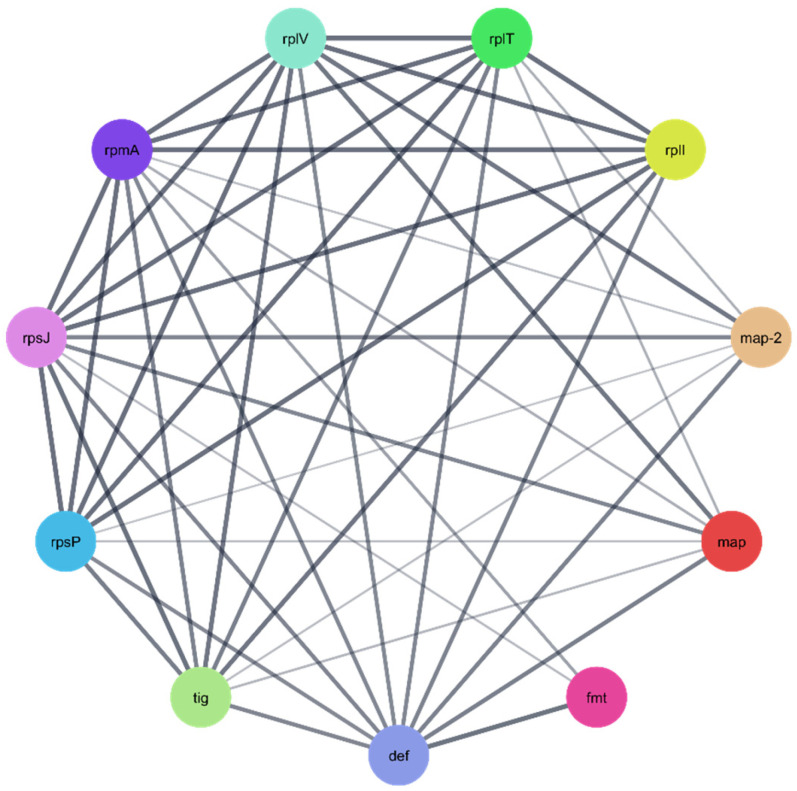
The PPI network of correlated *Pseudomonas aeruginosa* protein targets for the polypeptide deformylase Q9I7A8 [62].

**Figure 13:**
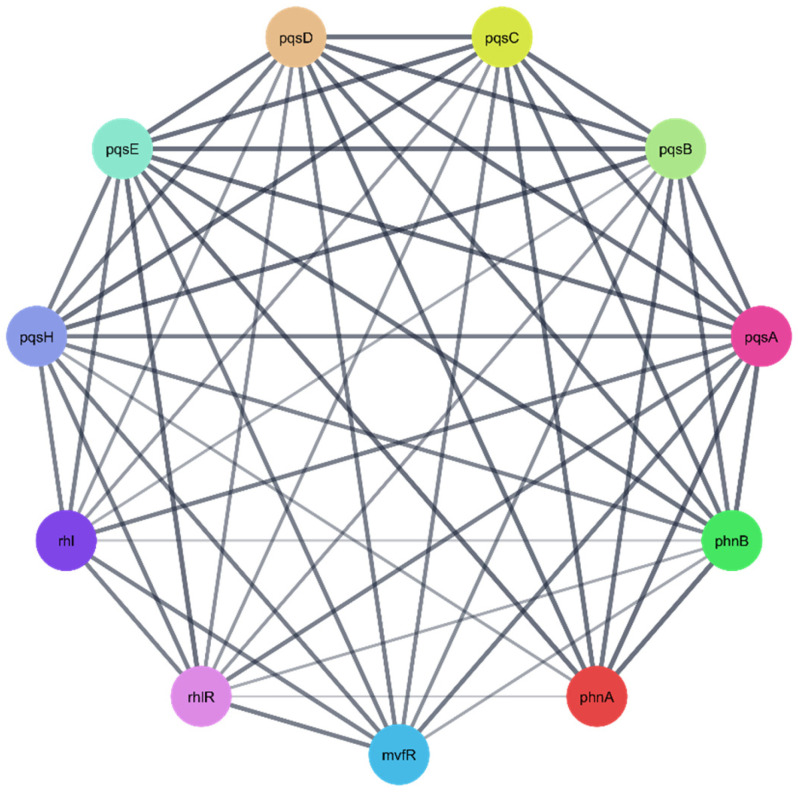
The PPI network of correlated *Pseudomonas aeruginosa* protein targets for the quinolone signal response protein P20581 (pqsE).

**Figure 14:**
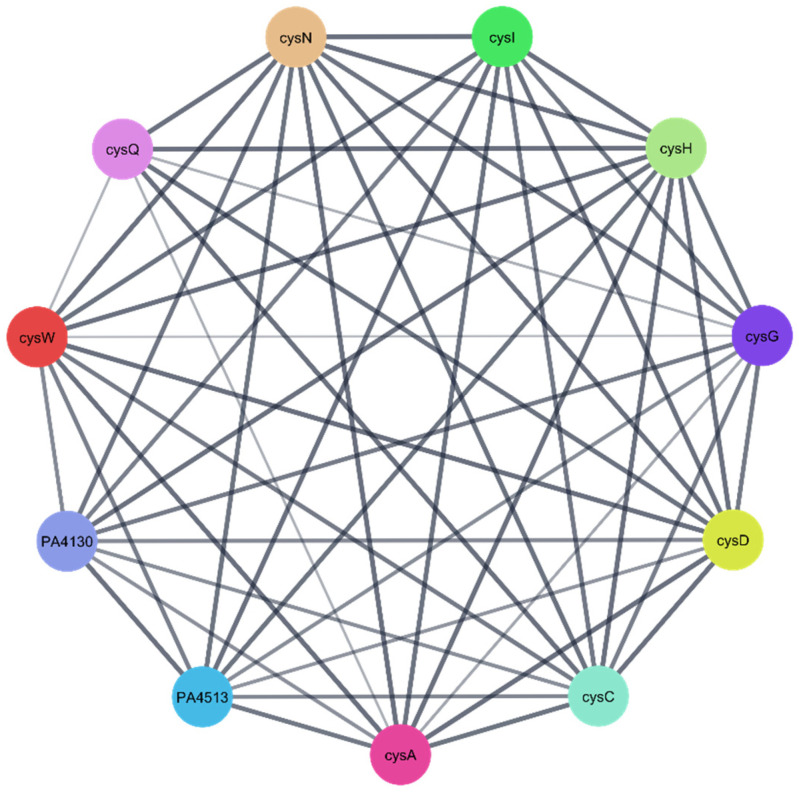
The PPI network of correlated *Pseudomonas aeruginosa* protein targets for the 3′-phosphoadenosine-5′-phosphosulfate reductase O05927 (cysH).

To further explore the biological relevance of the three identified *P. aeruginosa* protein targets (Table 4), protein–protein interaction (PPI) networks were constructed using the STRING database. In the resulting networks, each node represents a protein target, while each edge denotes a predicted or known interaction between proteins. The generated PPI networks highlighted the central role of these targets, suggesting that they function as key regulatory hubs—or “maestro” regulators—interacting with multiple other proteins within the *P. aeruginosa* proteome. This emphasizes their potential significance as therapeutic targets against *P. aeruginosa* (Figure 12, Figure 13 and Figure 14).

The three *Pseudomonas aeruginosa*-related proteins targets (Table 4) of compounds CID 72277 and CID 5281643 underwent enrichment analysis to understand their biological functions and possible pathways. Enrichment analyses were performed through Gene Ontology (GO) and the Kyoto Encyclopedia of Genes and Genomes (KEGG).

### 2.10. Gene Ontology (GO) Enrichment Analysis

**Figure 15:**
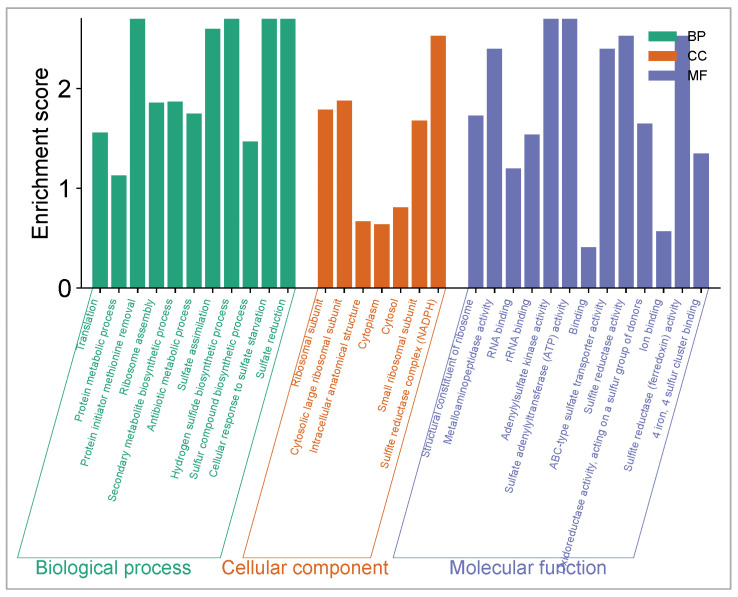
GO enrichment analysis, including biological process, cellular components, and molecular function for three *Pseudomonas aeruginosa*-related protein targets (Q9I7A8, P20581, and O05927) of compounds CID 72277 and CID 5281643.

GO enrichment analysis is a method for interpreting gene or protein sets by associating them with known biological functions, including biological processes [63], cellular components (CCs), and molecular functions (MFs) [60,64]. The results, illustrated by the histogram, show that the candidate targets are primarily involved in biological processes such as translation, ribosome assembly, and cellular response to sulfate starvation. In terms of molecular functions, the targets are associated with structural constituents of the ribosome, ATP binding activity, and sulfate reductase activity. Cellular component analysis indicates enrichment in the cytosol, NADPH-related structures, and ribosomal subunits (Figure 15).

### 2.11. KEGG Pathway Enrichment Analysis

**Figure 16:**
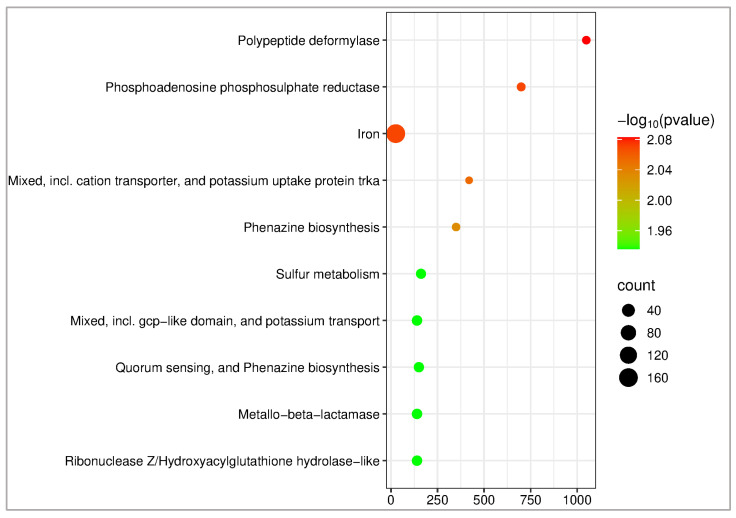
KEGG pathway enrichment analysis for three *Pseudomonas aeruginosa*-related protein targets (Q9I7A8, P20581, and O05927) of compounds CID 72277 and CID 5281643.

KEGG (Kyoto Encyclopedia of Genes and Genomes) enrichment analysis is used to map protein targets to relevant molecular pathways [60]. The analysis was performed to identify potential biological pathways associated with the predicted protein targets of the selected phytochemicals. The top 10 enriched pathways were selected based on enrichment scores and are visualized in Figure 16. The KEGG analysis revealed that the protein targets related to CID 72277 and CID 5281643 are predominantly associated with pathways involving polypeptide deformylase and phosphoadenosine phosphosulfate reductase, which represent the most significantly enriched functions.

### 2.12. Molecular Docking

In order to further verify the active ingredients, their potential targets, and their mechanistic role against *P. aeruginosa*, the most significant core targets (polypeptide deformylase and phosphoadenosine phosphosulfate reductase) based on KEGG pathways and other aforementioned results were selected for virtual screening docking simulation studies against the emerged two active metabolites of *Nephrolepis exaltata* extract (CID 72277 and CID 5281643) using MOE software.

**Figure 17:**
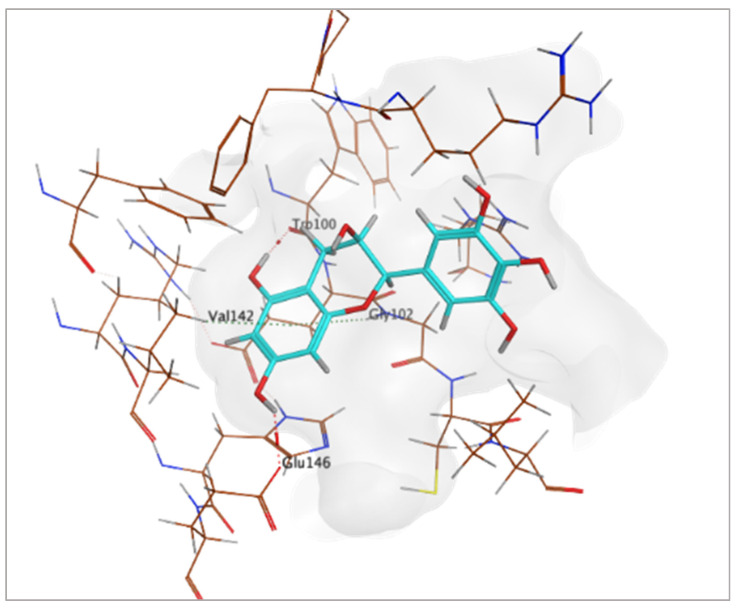
3D-representation of (CID 72277) against peptide deformylase, PDB IDs: 6JFF.

The findings demonstrated that both active metabolites interacted with the primary target, peptide deformylase (PDB ID: 6JFF). Among them, compound CID 72277 exhibited the strongest binding affinity, with a docking score of −6.0270 kcal/mol. Its binding mode involved two hydrogen bonds and two hydrophobic interactions with critical amino acid residues Trp100, Glu146, Gly102, and Val142 (Table 5 and Figure 17) [9]. Conversely, compound CID 5281643 displayed a moderate binding affinity, characterized by a docking score of −5.0920 kcal/mol and interactions including hydrogen bonds and hydrophobic contacts with residues Trp100, Glu146, and His145 (Table 5).

**Figure 18:**
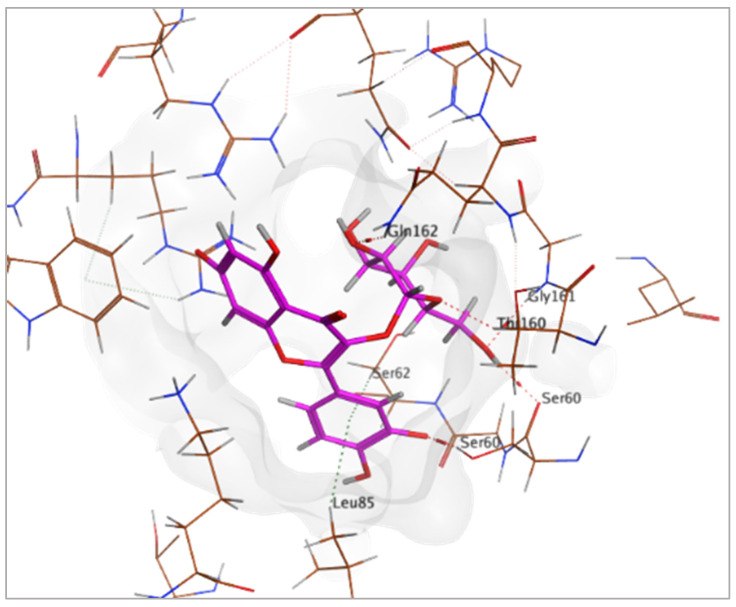
3D-representation of (CID 5281643) against adenosine 5′-phosphosulfate reductase, PDB IDs: 2GOY.

Regarding adenosine 5′-phosphosulfate reductase (PDB ID: 2GOY), CID 5281643 emerged as the more potent ligand, achieving a binding score of −7.1013 kcal/mol. Its interaction profile included hydrogen bonding, hydrophobic, and ionic interactions with key residues Gly161, Thr160, Ser60, Glu162, Leu85, Ser62, Arg242, and Arg145 (Table 5 and Figure 18). In contrast, CID 72277 exhibited comparatively weaker binding to this target, with a docking score of −5.6139 kcal/mol. Its binding was limited to residues Arg242 and Gly161 via hydrogen bonds (Table 5). These molecular docking results are consistent with the pharmacophore modeling predictions previously obtained from PharmMapper for both compounds.

### 2.13. Predicting the Role of MnO_2_-MgO BNPs in Antipseudomonal Bacterial Effect of Nephrolepis exaltata Extract

#### 2.13.1. ASKCOS Prediction

In this study, the ASKCOS platform (Automated Synthesis Knowledge Construction and Optimization System) was employed to predict the redox reaction products involved in the preparation of the nano-formulation derived from the interaction between MnO_2_-MgO BNPs and the selected bioactive compounds (CID 72277 and CID 5281643) (Table 6). ASKCOS (https://askcos.mit.edu/forward?tab=forward, accessed on 6 July 2025) is an open-source, machine learning-based software suite widely used in computer-aided synthesis planning (CASP). It is frequently cited in the fields of drug discovery, cheminformatics, and synthetic route design due to its ability to utilize extensive reaction datasets as well as predictive algorithms to propose feasible synthetic pathways [65]. In this context, ASKCOS facilitated the prediction of the likely redox transformations, providing essential insight into the chemical nature of the expected products, which were subsequently subjected to further computational evaluation and molecular docking studies.

#### 2.13.2. Molecular Docking Simulation

With respect to the outer membrane porin OprD of *P. aeruginosa* (PDB ID: 3SY7), the oxidized forms of CID 72277 and CID 5281643 exhibited enhanced binding affinities, suggesting efficient interaction with the porin channel. This implies the potential for successful intracellular delivery of the compounds via the MnO_2_-MgO-based nano-formulation, facilitating their penetration into the bacterial cell. Once internalized, the oxidized compounds are anticipated to be reduced back to their native bioactive forms by intracellular redox systems of *P. aeruginosa*, including NADH, glutathione, and phenazine-mediated pathways [66]. This mechanistic insight correlates with the observed enhancement in antibacterial activity, as demonstrated in the biological assays following nano-formulation of the NEME.

Molecular docking analysis revealed that compounds CID 72277 and CID 5281643 achieved binding scores of −4.6547 and −5.7701 kcal/mol., respectively. Their binding interactions involved key residues within the OprD binding pocket: CID 72277 formed hydrogen bonds and hydrophobic contacts with Ala199, Gly189, Gly188, Leu201, and Leu152, while CID 5281643 interacted with Ile187, Leu152, Gly189, and Leu201 (Table 7).

Collectively, the integration of the in silico approaches has provided crucial insights into the antimicrobial potential of *N. exaltata* extract, particularly against *Pseudomonas aeruginosa*. The computational screening via PASS Online revealed that several bioactives, especially epigallocatechin and hyperoside, possess strong antibacterial activity (Pa ≥ 0.5), supporting findings of the biological measures. Pharmacophore-based target prediction using PharmMapper identified bacterial protein targets specific to *P. aeruginosa*, including polypeptide deformylase, pqsE, and PAPS reductase. These proteins were further validated as central nodes in protein–protein interaction networks, indicating their potential role as key regulatory elements in bacterial viability. GO and KEGG enrichment analyses highlighted their involvement in critical biological functions such as translation and sulfate metabolism. Subsequent molecular docking confirmed strong interactions between these compounds and their predicted targets, with epigallocatechin displaying the highest affinity towards peptide deformylase, while hyperoside showed superior binding to PAPS reductase. Interestingly, formulating these compounds into MnO_2_–MgO-based nanoformulations further enhanced their binding to the outer membrane porin OprD, suggesting improved cellular uptake. The redox-responsive nature of the formulation allows intracellular reduction of the compounds to their active forms, thus amplifying their antibacterial potential.

## 3. Material and Methods

### 3.1. Chemicals and Reagents

Fresh leaves of *Nephrolepis exaltata* were purchased in September, 2024 from El-Minia garden. The plant was kindly identified by Dr. Kholoud Nagy, the assistant professor of botany, Faculty of Sciences, Minia University. Manganese acetate tetra hydrate (Mn(CH_3_COO)_2_·4H_2_O) and )Mg(NO_3_)_2_·6H_2_O), as sources of metal ions as well as NaOH, were purchased from Sigma-Aldrich^®^, Cairo, Egypt.

### 3.2. Preparation of Nephrolepis exaltata Methanol Extract (NEME)

Fresh leaves of *N. exaltata* were thoroughly rinsed twice with distilled water, dried with a towel, cut, and left to dry in shade for about 1 week. After complete dryness, the leaves (500 g) were powdered, macerated with 95% methanol, and concentrated using a Rotavap (Heidolph, Schwabach, Germany), which gave a crude extract (100 g) that was stored in the refrigerator at 4 °C [67].

### 3.3. UPLC/MS Analysis of Nephrolepis exaltata Extract

The UPLC/MS analysis for the *N. exaltata* extract was executed using a XEVO TQD triple quadruple instrument, Waters Corporation, Milford, MA, USA, with ESI-MS positive and negative ion acquisition modes using an ACQUITY UPLC—BEH C18 column (1.7 mm − 2.1 mm × 50 mm) with a flow rate of 0.2 mL/min, applying a gradient of water containing 0.1% formic acid and acetonitrile containing 0.1% formic acid [13,20,68,69,70].

### 3.4. Biosynthesis of MnO_2_-MgO BNPs of Nephrolepis exaltata Extract

The MnO_2_-MgO BNPs were prepared using different concentrations of salts compared to bimetallic biosynthesis [71]. A 10 mL (0.05 M) sample of Mn (CH_3_COO)_2_·4H_2_O and 10 mL (0.2 M) of Mg (NO_3_)_2_·6H_2_O) were mixed and then agitated for 20 min. Then, 80 mL of the formulated *Nephrolepis exaltata* extract was added and finally the PH was adjusted to 9.0. For optimizing the synthesis of MnO_2_-MgO BNPs, the reaction was conducted in a shaking incubator at 35 °C with continuous agitation at 150 rpm for approximately 24 h, resulting in a color change to black which suggests the successful bioformation of MnO_2_-MgO BNPs [36]. To ensure purity of the bimetallic nanoparticles, 5 washings with distilled water were employed to remove any loosely bound organic residues. This was followed by centrifugation at 10,000 rpm for 10 min. The precipitate was then oven-dried at 70 °C for 48 h.

### 3.5. Characterization of the Formed MnO_2_-MgO BNPs

The initial formation of MnO_2_-MgO BNPs was detected by the color change of the *NEE* from yellow to black. UV–visible spectroscopy (JENWAY 6305, Keison Products, Staffordshire, UK) was employed to measure the absorbance of the synthesized NP solution at wavelengths ranging from 200 to 700 nm to determine the maximum SPR. FTIR analysis (Cary-660 model, Agilent Technologies, Santa Clara, CA, USA) was conducted using the KBr pellet method at the wavenumber range of 400 to 4000 cm^−1^ to identify the chemical functional groups formed between the MnO_2_-MgO BNPs [72]. TEM with a JEM-2100 Plus, Jeol, Tokyo, Japan apparatus was employed to estimate the average dimensions and morphologies of the formed MnO_2_-MgO BNPs. DLS, using a Nano Zetasizer instrument from Malvern Panalytical, Malvern, England, was employed to measure the mean particle size distribution of the synthesized MnO_2_-MgO BNPs. An XRD-6000, Shimadzu, Kyoto, Japan was harnessed to estimate the crystal size and crystallinity of the bimetallic MnO_2_-MGO BNPs [8]. Scanning electron microscopy (SEM, ZEISS, EVO-MA10, Oberkochen, Germany) was used to examine the surface structure, while energy-dispersive X-ray spectroscopy (EDX, Bruker, Bremen, Germany) confirmed the elemental composition, purity, and distribution of the synthesized MnO_2_-MgO BNPs.

### 3.6. Evaluation of the Antimicrobial and Anti-Biofilm Activity

#### 3.6.1. Selection of the Isolates

The bacterial isolates and standard strains used in this study were obtained from the Bacteriology laboratory at the Botany and Microbiology Department, Faculty of Science, Al-Azhar University. These microorganisms included gram-negative species such as *Escherichia coli* and *Pseudomonas aeruginosa*, as well as the gram-positive species *Bacillus cereus*. The identification and antibiotic susceptibility testing were previously conducted using the VITEK2 system (BioMérieux^®^, Inc., Durham, NC, USA) [73]. In addition to these bacterial strains, standard strains of *Staphylococcus aureus* (ATCC-6538), *Klebsiella pneumoniae* (ATCC-9633), and *Candida albicans* (ATCC-10231) were employed for testing the antimicrobial activity and biofilm inhibition potency of MnO_2_-MgO BNPs.

#### 3.6.2. The Antimicrobial Activity

The antimicrobial activity of *Nephrolepis exaltata* extract, MnO_2_, MgO, and MnO_2_-MgO BNPs was assessed using Mueller Hinton Agar (Ministry of Home Affairs, New Delhi, India) for bacterial strains and Potato Dextrose Agar for *Candida albicans* [74]. Fresh 24 h cultures of tested microorganisms were inoculated onto the surface of the prepared MHA and PDA plates for bacterial and fungal testing, respectively. Wells of 6 mm diameter were made using a sterile cork borer and 100 µL of each test compound was carefully dispensed into separate wells. The plates were then left at 4 °C for 2 h to allow diffusion. The inoculated plates were subsequently incubated for 24 h at 37 °C for all bacterial strains and 48 h at 28 °C for *Candida albicans*. After the incubation period, inhibition zones were measured and recorded to evaluate the antimicrobial efficacy of each of the tested formulations [75].

#### 3.6.3. Determination of the MIC

The MIC values of MnO_2_-MgO BNPs were determined via the broth microdilution assay method reported by El-Didamony et al. [73]. Various concentrations of MnO_2_-MgO BNPs were prepared, and 100 µL of the tested preparations was added to sterile microtiter plate wells containing 100 µL of double-strength Mueller Hinton (MH) broth, all of which resulted in final concentrations of 1000, 500, 250, 125, 62.5, and 31.25 µg/mL. A microbial cell suspension (50 µL) adjusted to an OD equivalent to the 0.5 McFarland standard was introduced into all wells, except for the negative control. The positive control wells contained MH broth and bacterial suspension to assure the ability of the broth to support bacterial growth, while the other wells included MH broth to ensure sterility. The plates were incubated at 37 °C for 24 h. To detect bacterial growth, 30 µL of resazurin solution (0.02% wt./*v*) was inoculated, followed by overnight incubation. A color change indicated the microbial growth, whereas the absence of color change in sterile control wells confirmed the absence of contamination. The experiment was conducted in triplicate and the mean values were calculated [76].

#### 3.6.4. The Anti-Biofilm Assay

The anti-biofilm activity of MnO_2_-MgO BNPs was assessed using 96-well microtiter plates (flat-bottom, polystyrene) following the method reported by Sharaf, MH [77]. Each well was filled with 100 μL of MHB, inoculated with 10 μL of an overnight microbial culture suspension, then adjusted to an OD_620_ of 0.05 ± 0.02. To this mixture, 100 μL of the MnO_2_-MgO BNPs of NEE was added at different concentrations corresponding to ½ × MIC, ¼ × MIC, and 1/8 × MIC. The plates were then incubated at 37 °C for 48 h and the biofilms were fixed using absolute alcohol and stained with 0.1% (*w*/*v*) crystal violet solution for 30 min. Finally, the wells were air-dried, where 200 μL of 33% CH3COOH was added to dissolve the stained biofilm, followed by measuring the OD at 630 nm. The percentage inhibition of biofilm formation was calculated using the following equation:

(1) $$Biofilm\;inhibition\left(\%\right)=1-\frac{OD630\;of\;cells\;treated\;with\;different\;concentration\;of\;{MnO}_{2}-MgOBNPs\;extract}{OD630\;of\;non\;treated\;control}\times 100$$

### 3.7. Antioxidant Assays (DPPH and APTS Scavenging Activities)

The free radical-scavenging potential of MnO_2_-MgO BNPs was determined via the DPPH reagent assay [78]. The DPPH reagent was prepared by dissolving 8 mg of DPPH in 100 mL of methanol to give a concentration of 80 µg/mL. To determine the scavenging activity, 100 µL of the DPPH reagent was combined with 100 µL of the test sample in a 96-well microplate and incubated at room temperature for 30 min, and then the absorbance was recorded at 490 nm using an ELISA reader (TECAN, Grödig, Austria), with 100% methanol as the control. The DPPH scavenging activity was calculated using the following formula:

(2) $$DPPH\;scavenging\;activity=\frac{control\;absorbance-{MnO}_{2}-MgOBNPs\;absorbance}{control\;absorbance}\times 100$$

To evaluate the DPPH radical scavenging activity, different concentrations of MnO_2_-MgO BNPs (1000, 500, 250, 125, 62.5, 31.25, 15.62, and 7.81 µg/mL) were tested. The antioxidant activity of both the standard and MnO_2_-MgO BNPs was expressed as DPPH scavenging activity (%). The half-maximal inhibitory concentration (IC_50_) was determined to assess the effectiveness of the NPs as antioxidants. Additionally, the antioxidant activity of MnO_2_-MgO BNPs was further evaluated using the ABTS (2,2′-azino-*bis-*(3-ethylbenzothiazoline-6-sulfonic acid)) assay. This method followed the protocol established by Lee [79] with minor modifications:

(3) $$APTS\;scavenging\;activity=\frac{control\;absorbance-{MnO}_{2}-MgOBNPs\;extract\;absorbance}{control\;absorbance}\times 100$$

### 3.8. In Silico Biological Activity Predictions

The Way2Drug platform offers a suite of computational tools designed to assist researchers in predicting various pharmacological and toxicological properties of chemical compounds, including biological activity spectra, cytotoxicity, adverse drug reactions, mechanisms of action, and interactions with metabolic enzymes [80]. Among these tools is PASS Online (Prediction of Activity Spectra for Substances), which predicts the biological activity profiles of compounds based on structure–activity relationships derived from a database of over one million biologically characterized molecules. PASS provides a probabilistic assessment of biological activity, expressed as the probability of activity (Pa) and inactivity (Pi), achieving an average predictive accuracy of approximately 95% [81]. The chemical structures of compounds 1–20 were retrieved along with their PubChem IDs and SMILES (Supplementary Table S1) using the PubChem^®^ database (https://pubchem.ncbi.nlm.nih.gov/, accessed on 6 July 2025), then submitted to PASS Online in the SMILES format, and their predicted activities were recorded. Compounds with Pa values ≥ 0.5 were considered potentially active and selected for further evaluation, with particular interest given to those with 0.7 > Pa > 0.5, indicating moderate confidence in activity (Table S2). Additionally, AntiBac-Pred (http://www.way2drug.com/antibac/, accessed on 6 July 2025), an extension of the PASS platform, was employed to predict the antimicrobial spectrum of the compounds against a panel of 353 bacterial strains. The output is provided as a confidence ratio, where higher values correspond to a greater likelihood of antibacterial activity against specific microbial species.

### 3.9. Prediction of the Potential Protein Targets of the Annotated Compounds

The potential molecular targets of the annotated compounds were predicted using the PharmMapper server, an advanced pharmacophore-based web tool that utilizes reverse molecular docking to identify potential targets [82,83]. The compounds were uploaded in SDF format, where default parameters were applied. Specifically, conformer generation was enabled with a maximum of 300 conformations per compound, energy minimization was activated, and the target set used for pharmacophore mapping was the “Druggable Pharmacophore Models” database (version 2017, containing 16,159 models). For compounds predicted to exhibit antibacterial activity, targets associated with Pseudomonas species were selectively retrieved for further analysis.

Subsequent target validation and interaction analysis were performed using STRING database version 11.5 (https://string-db.org/, accessed on 6 July 2025), which compiles and integrates known and predicted protein–protein interactions [60]. The list of predicted targets from PharmMapper was input into STRING, specifying *Pseudomonas aeruginosa* as the organism of interest. A medium confidence threshold (interaction score ≥ 0.4) was applied.

### 3.10. Gene Ontology (GO) and Kyoto Encyclopedia of Genes and Genomes (KEGG) Enrichment Analysis

To gain deeper insights into the biological functions and associated pathways of the identified protein targets, functional enrichment analyses were conducted using Gene Ontology (GO) and the Kyoto Encyclopedia of Genes and Genomes (KEGG) databases. GO analysis categorizes gene functions into three domains: biological process [63], cellular component (CC), and molecular function (MF), providing a comprehensive understanding of gene roles. KEGG pathway analysis elucidates the biological pathways in which the target genes are involved. GO enrichment analysis was performed and visualized using STRING database, applying a false discovery rate (FDR) threshold of < 0.05 to ensure statistical significance. KEGG pathway enrichment was performed and visualized using the ShinyGO (version 0.76.3; http://bioinformatics.sdstate.edu/go/, accessed on 6 July 2025) and SRplot tools (https://www.bioinformatics.com.cn/en, accessed on 6 July 2025), facilitating the interpretation of key pathways associated with the predicted targets [60,64,84].

### 3.11. Molecular Docking

Molecular docking was performed between compounds CID 72277 (epigallocatechin) and CID 5281643 (hyperoside) and the core targets peptide deformylase and adenosine 5′-phosphosulfate reductase. The Molecular Operating Environment (MOE) 2019.0102 software was used for molecular docking simulation for the studying of binding affinity *N. exaltata* metabolites against target enzymes. The database of the active metabolites was drawn and prepared via energy minimization, hydrogen addition, and calculation of the partial charges. Finally, this prepared database was saved in the form of mdb extension. The target enzymes were retrieved from Protein Data Bank (www.rcsb.org) with PDB IDs: 6JFF and 2GOY. They were prepared and checked through automatic quick prepare order of MOE. The docking simulation was implemented via Amber10 Forcefield. Evaluation of ligand–protein complex interactions was afforded through visualization of poses and scoring function. Validation of docking studies was examined by the Root Mean Square Deviation (RMSD) values for co-crystalized ligand protein iso-zymes, (peptide deformylase, PDB IDs: 6JFF, RMSD:1.6367) and (adenosine 5′-phosphosulfate reductase, PDB IDs: 2GOY, RMSD:1.0805).

### 3.12. Predicting the Role of MnO_2_-MgO BNPs in Antipseudomonal Bacterial Effect of Nephrolepis exaltata Extract

The potential reaction products formed between the MnO_2_–MgO composites and the top-ranked bioactive constituents of the extract (CID 72277 and CID 5281643) were predicted using the ASKCOS online tool (https://askcos.mit.edu/, accessed on 6 July 2025). Subsequently, the resulting products were subjected to molecular docking studies against the high-resolution crystal structure of the *Pseudomonas aeruginosa* outer membrane protein OprD (PDB ID: 3SY7), a critical porin that regulates the transport of small molecules into and out of the bacterial cell. This protein is known to contribute to antibiotic resistance mechanisms, and structural insights into OprD can facilitate the development of therapeutic strategies to counter such resistance.

Molecular docking simulations were carried out using the Molecular Operating Environment (MOE) software. The active metabolites were sketched, energetically minimized, and structurally optimized by adding hydrogen atoms and calculating partial atomic charges. The processed ligands were stored in MOE’s proprietary database format (mdb). The OprD protein structure was retrieved from the Protein Data Bank (PDB ID: 3SY7) and prepared using MOE’s QuickPrep protocol. Docking procedures were executed using the Amber10 force field. Ligand–protein interactions were evaluated based on binding poses and scoring functions. The reliability of the docking protocol was confirmed by calculating the Root Mean Square Deviation (RMSD) between the docked and co-crystallized ligand poses, yielding an RMSD value of 1.3874 Å, which indicates acceptable accuracy.

### 3.13. Statistical Analysis

The statistical analysis of the obtained data was visualized by GraphPad Prism version (8.0.2). To make comparison of the differences between groups, an independent t-test was employed. The analysis was conducted using the Sorensen (Bray–Curtis) method for group linkage, which enabled the clustering of similar groups based on their characteristics. ANOVA (Analysis of Variance) and Tukey’s post hoc test were applied for multiple comparisons.

## 4. Conclusions

The current study investigated the antimicrobial and anti-biofilm activities of greenly synthesized MnO_2_-MgO BNPs against several tested pathogens, where the results revealed a concentration-dependent reduction in their biofilm formation. Accordingly, *Nephrolepis exaltata* nanoparticles may act as a natural source for new antimicrobial drugs with potent biofilm inhibition activity. Future research is encouraged in order to isolate the main components responsible for the observed activities. The progressive challenge of antibiotic resistance necessitates the evolution of standby strategies to ensure good health, safety, and improved quality of life. This study successfully synthesized and characterized MnO_2_-MgO bimetallic nanoparticles using *Nephrolepis exaltata* as a sustainable reducing agent, which correlates to green chemistry principles. Such a cost-effective and eco-friendly approach underscores the potential of MnO_2_-MgO BNPs in combating *Psudomonas aeruginosa* strains, which are a major public health threat associated with blood and lung infections, while aligning with global sustainability goals.

The greenly synthesized MnO_2_-MgO BNPs exhibited superior purity and distinctive morphologies, with a dose-dependent anti-biofilm inhibition of *P. aeruginosa* strains. The findings highlight the efficiency of eco-friendly MnO_2_-MgO BNPs as potent antibacterial agents, presenting a foundation for future research to elucidate their mechanisms and explore their broader applicability across multidisciplinary domains, potentially improving public health and sustainable practices.

## Figures and Tables

**Table 1:** The tentatively identified components from *Nephrolepis exaltata* (Fujar) through UPLC/MS.

No.	Component	Molecular Formula	Chemical Class	R_t_ (min.)	[M − H]^−^ *m*/*z*	[M + H]^+^ *m*/*z*	Area %	Ref.
1	Tetrahydroxy trimethoxy dihydroxyflavone	C_19_H_18_O_10_	Flavonoid	0.81	377	381	12.03 (18.13)	[13,14]
2	Kaempferol-*di*-hexoside	C_42_H_45_O_22_	Flavonoid	6.04	609	-	4.75	[15]
3	Kaempferol-hexoside	C_21_H_20_O_11_	Flavonoid	6.64	447	-	2.82	[15]
4	Afzelin *	C_21_H_20_O_10_	Flavonoid	7.08	431	-	2.23	[2]
5	Quercetin-hexoside	C_21_H_20_O_12_	Flavonoid	7.65	463	-	8.15	[15]
6	Kaempferol-hexuronoide	C_27_H_30_O_16_	Flavonoid	8.05	459	-	0.58	[15]
7	Unidentified	-	-	8.61	-	162 or 194 or 217	6.17	-
8	Ethyl palmitate	C_18_H_36_O_2_	Fatty acid	11.52	-	285	2.04	[16]
9	Unidentified	-	-	14.35	-	277 or 353 or 393	0.75	-
10	9-*oxo*-Octadecadienoic acid	C_18_H_32_O_3_	Fatty acid	14.41	293	-	20.80	[17,18]
11	Myricetin	C_15_H_10_O_8_	Flavonoid	15.48	-	319	0.69	[16]
12	Myricetin-pentosyl pentoside	C_27_H_30_O_16_	Flavonoid	15.96	609	611	7.69 (1.49)	[16]
13	Chlorogenic acid	C_16_H_18_O_9_	Phenolic acid	16.44	353	355	5.49 (6.40)	[13,14,19]
14	Trihydroxy germacrenolide	-	Sesquiterpene lactone	16.94	-	285	2.75	[16]
15	*p*-Coumaroyl-hexoside	C_15_H_14_N_2_O_3_	Phenolic acid	17.36	325	-	11.43	[16]
16	Caffeic acid hexoside	C_9_H_8_O_4_	Phenolic acid	19.13	341	347	6.22 (2.75)	[14,20]
17	Carnosic acid	C_20_H_28_O_4_	Phenolic acid	19.99	-	333	2.49	[14,21]
18	Trigalloyl hexoside	C_27_H_24_O_18_	Tannin	20.99	-	637	1.10	[22]
19	Malvidin	C_17_H_15_O_7_^+^	Anthocyanin	21.24	-	331	0.61	[23]
20	Glycitein-hexouronide	C_22_H_20_O_11_	Flavonoid	22.73	459	461	0.29 (17.92)	[24,25]
21	Benzoyl caffeic acid rutinoside	-	Phenolic acid	26.00	591	-	0.29	[18]
22	Unidentified	-	-	27.62	-	613	2.61	-
23	Unidentified	-	-	27.84	-	647 or 613	2.41	-
24	Lutein (hydroxycarotenoid)	C_40_H_56_O_2_	Carotenoid	28.05	-	568	2.84	[23]
25	Hyperoside	C_21_H_20_O_12_	Flavonoid	28.28	-	465	3.67	[19]
26	Syringaresinol−acetyl hexose	C_30_H_37_O_14_	Iridoid	28.41	621	757	1.60 (2.85)	[26]
27	Unidentified	-	-	28.89	-	445	4.48	-
28	Galloyl-HHDP	C_35_H_16_O_21_	Tannin	29.28	481	-	0.56	[27]
29	Methoxy ursolic acid	C_31_H_51_O_4_	Triterpene	29.54	-	487	2.83	[28]
30	Oleanolic acid *	C_30_H_48_O_3_	Triterpene	29.71	457	-	2.78	[29]
31	Methoxy benzoic acid (*p*-Anisic acid)	C_8_H_8_O_3_	Phenolic acid	30.07	313	315	1.05 (2.28)	[16]
32	Caffeoyl quinic acid dimer	C_32_H_36_O_18_	Phenolic acid	30.72	707	-	2.41	[30]
33	Unidentified	-	-	31.11	265	-	6.04	-
34	Epigallocatechin *	C_15_H_14_O_7_	Tannin	31.34	305	307	0.76 (2.41)	[2]
	% identification							
	−ve mode				91.93%			
	+ve mode				55.12%			

* For compounds previously reported from the genus *Nephrolepis*. Area % between brackets is for the compounds in ESI positive mode.

**Table 2:** Antimicrobial activity of *Nephrolepis exaltata* extract, Mn nanoparticles, Mg nanoparticles, and MnO_2_-MgO BNPs.

Microbial Strain	Diameter of Inhibition Zone (mm)				
	*Nephrolepis exaltata* Extract	Magnesium Nanoparticle	Manganese Nanoparticle	MnO_2_-MgO BNPs	Chloramphenicol/ Clotrimazole
*Pseudomonas aeruginosa*	16 ± 0.5	11.8 ± 0.4	12 ± 0.5	26 ± 0.5	15.3 ± 0.3
*Klebsiella pneumonia* (ATCC-9633)	15 ± 0.5	17.1 ± 0.4	16 ± 0.5	22.6 ± 0.3	24.5 ± 0.2
*Staphylococcus aureus* (ATCC-6538)	15.3 ± 0.3	16 ± 0.5	14.6 ± 0.3	19.3 ± 0.8	19 ± 0.5
*Escherichia coli*	0	14.6 ± 0.3	12.1 ± 0.4	17 ± 0.5	21.3 ± 0.6
*Bacillus cereus*	21 ± 0.5	17.5 ± 0.2	22.6 ± 0.3	16 ± 0.5	25.6 ± 0.3
*Candida albicans* (ATCC-10231)	0	12.6 ± 0.3	0	13.3 ± 0.3	20 ± 0.5

**Table 3:** MIC values of MnO_2_-MgO BNPs against the different tested strains.

Microbial Strain	MIC Values of MnO_2_-MgO BNPs (µg/mL)
*Pseudomonas aeruginosa*	250 μg/mL
*Klebsiella pneumonia* (ATCC-9633)	500 μg/mL
*Staphylococcus aureus* (ATCC-6538)	500 μg/mL
*Escherichia coli*	500 μg/mL
*Bacillus cereus*	500 μg/mL
*Candida albicans* (ATCC-10231)	250 μg/mL

**Table 4:** Predicted refined results of PharmMapper for compounds CID 72277 and CID 5281643.

Compound Pubchem CID	*Pseudomonas aeruginosa* Related Proteins	Uniprot ID
72277 (epigallocatechin)	Polypeptide deformylase	Q9I7A8
	Quinolone signal response protein (pqsE)	P20581
5281643 (hyperoside)	3′-phosphoadenosine-5′-phosphosulfate reductase (PAPS reductase)	O05927

**Table 5:** Docking results of compounds CID 72277 and CID 5281643 against target proteins peptide deformylase (PDB ID: 6JFF) and adenosine 5′-phosphosulfate reductase (PDB ID: 2GOY).

Active Metabolites PubChem Id (CID)	Target Protein (Isozyme)	Binding Score kcal/mol	Key Amino Acid Residues	Type of Binding	2D Representation
CID 72277	PDB IDs: 6JFF	−6.0270	Trp100, Glu146, Gly102, Val142	H-Bond H-Bond Hydrophobic Hydrophobic	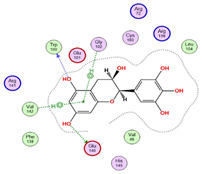
CID 5281643	PDB IDs: 6JFF	−5.0920	Trp100 Glu146, His145	H-Bond H-Bond Hydrophobic	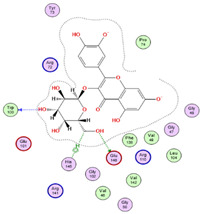
CID 72277	PDB IDs: 2GOY	−5.6139	Arg242, Gly161	H-Bond H-Bond	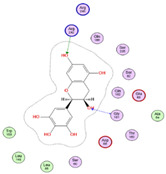
CID 5281643	PDB IDs: 2GOY	−7.1013	Gly161, Thr160, Ser60, Glu162, Leu85, Ser62, Arg242, Arg145	H-Bond H-Bond 2x H-Bond H-Bond Hydrophobic Hydrophobic Ionic Ionic	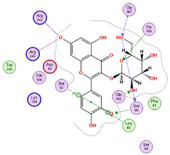

**Table 6:** ASKCOS prediction of redox transformations of compounds CID 72277 and CID 5281643.

CID 72277 Oxidized Form	CID 5281643 Oxidized Form
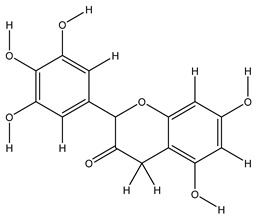	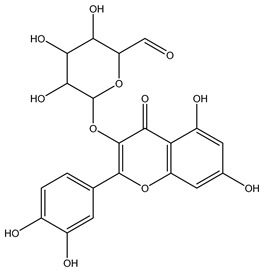

**Table 7:** Docking results of compounds CID 72277 and CID 5281643 against *Pseudomonas aeruginosa* outer membrane protein OprD (PDB ID: 3SY7).

Active Metabolites PubChem Id (CID)	Target Protein (Isozyme)	Binding Score kcal/mol	Key Amino Acid Residues	Type of Binding	2D Representation
CID 72277	PDB IDs: 3SY7	−4.6547	Ala199 Gly189, Gly188, Leu201 Leu152	H-Bond H-Bond H-Bond H-Bond Hydrophobic	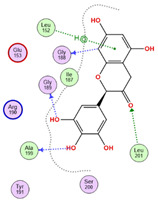
CID 5281643	PDB IDs: 3SY7	−5.7701	Ile187 Leu152 Gly189, Leu201	2x H-Bond H-Bond H-Bond 2x Hydrophobic	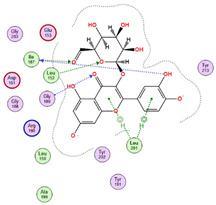

## Data Availability

All data are available in the manuscript as well as the Supplementary Materials.

## Supplementary Materials

The following supporting information can be downloaded at: https://www.mdpi.com/article/10.3390/ph18091262/s1; Table S1: SMILES and pubchem CID of active metabolites of Nephrolepis exaltata extract.; Table S2: Predicted bacterial effect of extract metabolites; Table S3: PharmMapper predicted protein targets for CID 72277.; Table S4: PharmMapper predicted protein targets for CID 5281643.; Table S5: Filtered predicted bacterial 80 protein targets for CID 72277.; Table S6: Filtered predicted bacterial 77 protein targets for CID 5281643.

## Abbreviations

Rotavap	Rotary evaporator
MnO_2_-MgO BNPs	Manganese dioxide-Magnesium oxide bimetallic Nanoparticles
NEE	*Nephrolepis exaltata* extract
SPR	Surface Plasmon Resonance
TEM	Transmission electron microscope
FTIR	Fourier transform infrared spectroscopy
DLS	Dynamic Light Scattering
SEM	Scanning electron microscope
MHA	Mueller Hinton Agar
PDA	Potato Dextrose Agar
OD	Optical density
MIC	Minimum inhibitory concentration
IC_50_	Inhibitory concentration of 50% of the microorganisms
FCC	Face-centered cubic
XRD	X-ray diffraction
EDX	Energy-Dispersive X-ray
