# Supplementary material for: Sustainable MnO2/MgO Bimetallic Nanoparticles Capped with Sword Fern Methanol Extract Attain Antioxidant/Anti-Biofilm Potential: A UPLC-ESI/LC/MS and Network Pharmacology-Supported Study
Source: Pharmaceuticals (Basel). 2025 Aug 25;18(9):1262. doi: 10.3390/ph18091262 (PMC12472329; doi:10.3390/ph18091262)
Supplement: Supplementary file 1 [file pharmaceuticals-18-01262-s001.zip › pharmaceuticals-3772397-supplementary.pdf]

Supplementary material

Sustainable MnO2/MgO Bimetallic Nanoparticles Capped with Sword Fern Methanol Extract Attain Antioxidant/Anti-biofilm Potential: A UPLC-ESI/LC/MS and Network Pharmacology-supported Study

Table S1: SMILES and pubchem CID of active metabolites of Nephrolepis exaltata extract.

**Component**	**smiles**	**Pub CID**
Tetrahydroxy trimethoxy dihydroxyflavone	C1=CC(=C(C=C1/C=C/C(=O)OC2C(C(C(C(O2)CO)O)O)O)O)O	6124135
Kaempferol-*di*-hexoside	C[C@H]1[C@@H]([C@H]([C@H]([C@@H](O1)OC[C@@H]2[C@H]([C@@H]([C@H]([C@@H](O2)OC3=C(OC4=CC(=CC(=C4C3=O)O)O)C5=CC=C(C=C5)O)O)O)O)O)O)O	5318767
Kaempferol hexoside	C1=CC(=CC=C1C2=C(C(=O)C3=C(C=C(C=C3O2)OC4C(C(C(C(O4)CO)O)O)O)O)O)O	5480982
Afzelin	C[C@H]1[C@@H]([C@H]([C@H]([C@@H](O1)OC2=C(OC3=CC(=CC(=C3C2=O)O)O)C4=CC=C(C=C4)O)O)O)O	5316673
Quercetin-hexoside	C1C(C(C(OC1OC2=C(OC3=CC(=CC(=C3C2=O)O)OC4C(C(C(C(O4)CO)O)O)O)C5=CC(=C(C=C5)O)O)O)O)O	133053374
Ethyl palmitate	CCCCCCCCCCCCCCCC(=O)OCC	12366
9-*oxo*-Octadecadienoic acid	CCCCC/C=C\C=C\C(=O)CCCCCCCC(=O)O	9839084
Myricetin-pentosyl pentoside	C1C(C(C(C(O1)OC2=C(OC3=CC(=CC(=C3C2=O)O)O)C4=CC(=C(C(=C4)O)O)O)O)O)O	21477996

Chlorogenic acid	C1[C@H]([C@H]([C@@H](C[C@@]1(C(=O)O)O)OC(=O)/C=C/C2=CC(=C(C=C2)O)O)O)O	1794427
Germacrene D	C/C/1=C\CCC(=C)/C=C/[C@@H](CC1)C(C)C	5317570
p-Coumaroyl-hexoside	CCCCCCCCCCCCCCCC(=O)O[C@H]1CC[C@@]2([C@H]3CC[C@]4([C@H](C3CCC2C1)CC[C@@H]4[C@H](C)CCC(CC)C(C)C)C)C	157010309
Caffeic acid hexoside	C1=CC(=C(C=C1C=CC(=O)O)O)O	2518
Carnosic acid	CC(C)C1=C(C(=C2C(=C1)CC[C@@H]3[C@@]2(CCCC3(C)C)C(=O)O)O)O	65126
Lutein (hydroxycarotenoid)	CC1=C(C(C[C@@H](C1)O)(C)C)/C=C/C(=C/C=C/C(=C/C=C/C=C(\C)/C=C/C=C(\C)/C=C/[C@H]2C(=C[C@@H](CC2(C)C)O)C)/C)/C	5281243
Hyperoside	C1=CC(=C(C=C1C2=C(C(=O)C3=C(C=C(C=C3O2)O)O)O[C@H]4[C@@H]([C@H]([C@H]([C@H](O4)CO)O)O)O)O)O	5281643
Methoxy ursolic acid	C[C@@H]1CC[C@@]2(CC[C@@]3(C(=CC[C@H]4[C@]3(CC[C@@H]5[C@@]4(CC[C@@H](C5(C)C)OC)C)C)[C@@H]2[C@H]1C)C)C(=O)O	90477731
Oleanolic acid	C[C@]12CC[C@@H](C([C@@H]1CC[C@@]3([C@@H]2CC=C4[C@]3(CC[C@@]5([C@H]4CC(CC5)(C)C)C(=O)O)C)C)(C)C)O	10494
Methoxy benzoic acid (*p*-Anisic acid)	COC1=CC=C(C=C1)C(=O)O	7478
Caffeoyl quinic acid	C1[C@H](C([C@@H](CC1(C(=O)O)OC(=O)/C=C/C2=CC(=C(C=C2)O)O)O)O)O	10155076
Epigallocatechin	C1[C@H]([C@H](OC2=CC(=CC(=C21)O)O)C3=CC(=C(C(=C3)O)O)O)O	72277

Table S2: Predicted bacterial effect of extract metabolites (Pa ≥ 0.5)

	Name	Confidence	ChEMBL ID
6124135	Clostridium ramosum	0.7159	CHEMBL614971
6124135	Yersinia pestis	0.6631	CHEMBL614597
6124135	Actinomyces meyeri	0.6447	CHEMBL612289
6124135	Clostridium cadaveris	0.6155	CHEMBL614970
6124135	RESISTANT Acinetobacter pittii	0.6126	CHEMBL3140321
6124135	Mycobacterium mageritense	0.585	CHEMBL612959
6124135	Streptococcus sanguinis	0.5804	CHEMBL612314
6124135	RESISTANT Mycobacterium ulcerans	0.5792	CHEMBL612965
6124135	Staphylococcus lugdunensis	0.5158	CHEMBL613303
6124135	Nocardia transvalensis	0.5044	CHEMBL613234
5318767	RESISTANT Mycobacterium ulcerans	0.589	CHEMBL612965
5318767	Mycobacterium mageritense	0.5611	CHEMBL612959
5318767	RESISTANT Acinetobacter pittii	0.5347	CHEMBL3140321
5318767	Clostridium ramosum	0.525	CHEMBL614971
5318767	Clostridium cadaveris	0.5036	CHEMBL614970
5318767	Streptococcus sanguinis	0.503	CHEMBL612314
5318767	Streptococcus pneumoniae R6	0.4899	CHEMBL2366794
5318767	RESISTANT Chlamydia trachomatis	0.4711	CHEMBL614606
5480982	Clostridium ramosum	0.6575	CHEMBL614971
5480982	RESISTANT Acinetobacter pittii	0.594	CHEMBL3140321
5480982	RESISTANT Mycobacterium ulcerans	0.5937	CHEMBL612965
5480982	Actinomyces meyeri	0.5835	CHEMBL612289
5480982	Clostridium cadaveris	0.5815	CHEMBL614970
5480982	Mycobacterium mageritense	0.5796	CHEMBL612959
5480982	Yersinia pestis	0.5427	CHEMBL614597
5480982	RESISTANT Burkholderia pseudomallei	0.5371	CHEMBL3140323

5480982	Nocardia transvalensis	0.5266	CHEMBL613234
5480982	Staphylococcus lugdunensis	0.4835	CHEMBL613303
5480982	RESISTANT Staphylococcus aureus subsp. aureus RN4220	0.4824	CHEMBL2366906
5316673	RESISTANT Mycobacterium ulcerans	0.6083	CHEMBL612965
5316673	Mycobacterium mageritense	0.5679	CHEMBL612959
5316673	RESISTANT Chlamydia trachomatis	0.5476	CHEMBL614606
5316673	Streptococcus pneumoniae R6	0.5434	CHEMBL2366794
5316673	RESISTANT Acinetobacter pittii	0.5275	CHEMBL3140321
5316673	Clostridium ramosum	0.5162	CHEMBL614971
5316673	RESISTANT Propionibacterium acnes	0.512	CHEMBL612639
5316673	Clostridium cadaveris	0.5083	CHEMBL614970
5316673	RESISTANT Staphylococcus aureus subsp. aureus RN4220	0.5067	CHEMBL2366906
5316673	RESISTANT Staphylococcus simulans	0.4851	CHEMBL612425
133053374	Clostridium ramosum	0.5586	CHEMBL614971
133053374	RESISTANT Mycobacterium ulcerans	0.5533	CHEMBL612965
133053374	Mycobacterium mageritense	0.5511	CHEMBL612959
133053374	RESISTANT Acinetobacter pittii	0.5491	CHEMBL3140321
133053374	Clostridium cadaveris	0.523	CHEMBL614970
133053374	Streptococcus pneumoniae R6	0.4702	CHEMBL2366794
133053374	Nocardia transvalensis	0.4622	CHEMBL613234
133053374	Actinomyces meyeri	0.4607	CHEMBL612289
12366	Streptococcus viridans	0.4959	CHEMBL612332
9839084	Streptococcus viridans	0.7217	CHEMBL612332
21477996	Streptococcus pneumoniae R6	0.5318	CHEMBL2366794
1794427	RESISTANT Chlamydia trachomatis	0.5095	CHEMBL614606
5317570	Prevotella oralis	0.4709	CHEMBL612687
2518	Streptococcus viridans	0.498	CHEMBL612332

65126	Bacillus subtilis subsp. subtilis str. 168	0.4919	CHEMBL613315
5281243	RESISTANT Staphylococcus simulans	0.5518	CHEMBL612425
5281243	Staphylococcus simulans	0.5344	CHEMBL612425
5281643	Streptococcus pneumoniae R6	0.826	CHEMBL2366794
5281643	Clostridium ramosum	0.6427	CHEMBL614971
5281643	RESISTANT Acinetobacter pittii	0.5842	CHEMBL3140321
5281643	RESISTANT Mycobacterium ulcerans	0.5818	CHEMBL612965
5281643	Clostridium cadaveris	0.5725	CHEMBL614970
5281643	Mycobacterium mageritense	0.5695	CHEMBL612959
5281643	Actinomyces meyeri	0.5677	CHEMBL612289
5281643	Nocardia transvalensis	0.5172	CHEMBL613234
5281643	RESISTANT Burkholderia pseudomallei	0.5086	CHEMBL3140323
5281643	Yersinia pestis	0.4811	CHEMBL614597
5281643	Staphylococcus lugdunensis	0.4665	CHEMBL613303
5281643	RESISTANT Staphylococcus aureus subsp. aureus RN4220	0.4544	CHEMBL2366906
5281643	Pseudomonas fluorescens	0.4532	CHEMBL612500
10494	Staphylococcus lugdunensis	0.5027	CHEMBL613303
10494	Lactobacillus plantarum	0.457	CHEMBL614973
7478	Yersinia pestis	0.8524	CHEMBL614597
7478	Dialister micraerophilus	0.6068	CHEMBL615038
7478	Dialister pneumosintes	0.6068	CHEMBL615039
7478	Dialister propionicifaciens	0.5917	CHEMBL615040
7478	RESISTANT Staphylococcus simulans	0.5769	CHEMBL612425
7478	Actinomyces meyeri	0.5632	CHEMBL612289
7478	Porphyromonas asaccharolytica	0.5468	CHEMBL615058
7478	Clostridium ramosum	0.5423	CHEMBL614971
7478	Prevotella disiens	0.5421	CHEMBL612235
7478	Parabacteroides merdae	0.5328	CHEMBL615057

7478	RESISTANT Helicobacter pylori	0.527	CHEMBL612600
7478	Staphylococcus lugdunensis	0.5121	CHEMBL613303
7478	Bacteroides stercoris	0.5061	CHEMBL614750
7478	Bacteroides uniformis	0.5051	CHEMBL612622
7478	Staphylococcus sciuri	0.4966	CHEMBL613150
7478	Prevotella oralis	0.4905	CHEMBL612687
7478	RESISTANT Clostridium perfringens	0.4694	CHEMBL614967
7478	RESISTANT Clostridium paraputrificum	0.455	CHEMBL615027
7478	RESISTANT Clostridium septicum	0.455	CHEMBL614968
7478	Clostridium innocuum	0.4548	CHEMBL614761
72277	Pseudomonas fluorescens	0.704	CHEMBL612500
72277	Listeria monocytogenes	0.4768	CHEMBL614974

Table S3: PharmMapper predicted protein targets for CID 72277

Name	Class	Uniprot id
Hyaluronate lyase	NONE	Q54873
Putative esterase HI1161	NONE	P45083
UPF0425 pyridoxal phosphate-dependent protein MJ0158	NONE	Q57622
Protein unc-45 homolog A	NONE	Q9H3U1
Major allergen Equ c 1	NONE	Q95182
Gag-Pro-Pol polyprotein	NONE	P03362
Thymocyte selection-associated high mobility group box protein TOX	NONE	Q66JW3
Protein C-ets-1	NONE	P27577
Leukemia inhibitory factor receptor	SIGNALING PROTEIN/CYTOKINE	LIFR_HUMAN
Bromodomain-containing protein 7	NONE	Q9NPI1
Periplasmic [NiFe] hydrogenase small subunit	NONE	P12943
Vacuolar protein sorting-associated protein 4	NONE	P52917
5-3 exoribonuclease 2	NONE	P40848

Acyl-coenzyme A oxidase 1, peroxisomal	NONE	P07872
Genome polyprotein	NONE	P13418
Penton protein	NONE	P03276
UPF0130 protein AF_2059	NONE	O28220
Nuclear pore complex protein Nup214	NONE	P35658
Beta-galactoside-specific lectin 4	NONE	Q6ITZ3
Siroheme synthase	NONE	P25924
Eukaryotic translation initiation factor 4E	NONE	P07260
Probable ATP-dependent RNA helicase DDX58	HYDROLASE	DDX58_HUMAN
Translation initiation factor eIF-2B subunit alpha	NONE	Q14232
Uncharacterized protein ypfI	NONE	P76562
Cytochrome b-c1 complex subunit 1, mitochondrial	NONE	P31800
Uncharacterized protein ykuL	NONE	O31698
Phycoerythrocyanin alpha chain	NONE	P00309
Calpain-9	HYDROLASE	CAN9_HUMAN
Pre-mRNA-splicing factor RBM22	NONE	Q9NW64
Arachidonate 12-lipoxygenase, 12S-type	NONE	P18054
Gag polyprotein	NONE	P03336
Salivary lipocalin	NONE	P81608
Flotillin-2	NONE	Q60634
Aspartate carbamoyltransferase regulatory chain	NONE	Q58801
Phycocyanobili	NONE	Q93TN0
H-2 class I histocompatibility antigen, K-B alpha chain	NONE	P01901
Isovaleryl-CoA dehydrogenase, mitochondrial	NONE	P26440
Acetate kinase	NONE	P38502
Insulin-like growth factor 1 receptor	TRANSFERASE	IGF1R_HUMAN
Signal transducer and activator of transcription 5A	NONE	P42230
Putative acyl-CoA thioester hydrolase ybhC	NONE	P46130

RNA polymerase II subunit A C-terminal domain phosphatase	NONE	Q9P376
GMP reductase	NONE	Q81JJ9
Circadian clock protein kinase kaiC	NONE	Q79PF4
Guanine nucleotide-binding protein subunit beta-like protein	NONE	O24456
Protein translocase subunit secA	NONE	P28366
Enhancer of mRNA-decapping protein 3	NONE	Q9VVI2
Glutamate-1-semialdehyde 2,1-aminomutase	NONE	Q5SJS4
Nicotinate-nucleotide pyrophosphorylase [carboxylating]	NONE	P43619
RNA-directed RNA polymerase lambda-3	NONE	P17378
D-tyrosyl-tRNA(Tyr) deacylase	NONE	O57774
Retinoic acid receptor gamma	GENE REGULATION	RARG_HUMA N
Adenylate kinase	NONE	Q5SHQ9
Cellular tumor antigen p53	NUCLEAR PROTEIN	P53_HUMAN
5-methyltetrahydropteroyltriglutamate--homocysteine methyltransferase	NONE	Q8CWX6
Sulfatase-modifying factor 1	NONE	Q8NBK3
Aspartate aminotransferase, cytoplasmic	NONE	P00503
YojF protein	NONE	O31858
Transposon Tn7 transposition protein tnsA	NONE	P13988
Protein farnesyltransferase/geranylgeranyltransferase type-1 subunit alpha	NONE	Q04631
Glutaminase	NONE	Q5KY26
Methylthioribose-1-phosphate isomerase	NONE	Q9X013
DNA protection during starvation protein	NONE	P0C558
Myelin P2 protein	NONE	P0C6G6
Arylphorin	NONE	Q7Z1F8
Protein MJ0882	NONE	Q58292
Kinetochore protein SPC25	NONE	P40014
Cleavage and polyadenylation specificity factor subunit 3	HYDROLASE, RNA BINDING PROTEIN	CPSF3_HUMA N
Malate dehydrogenase [NADP], chloroplast	NONE	P46489
Cysteine protease avirulence protein avrPphB	NONE	Q52430

Heat-inducible transcription repressor hrcA	NONE	Q9WZV5
Alpha-amylase type A isozyme	NONE	P00693
L-2-hydroxyisocaproate dehydrogenase	NONE	P14295
Ig gamma-1 chain C region	NONE	P01857
Tyrosine-protein kinase Lyn	NONE	P25911
DNA-binding protein SATB2	DNA BINDING PROTEIN	SATB2_HUMA N
Cytochrome b	NONE	Q02761
Serine/threonine-protein kinase D2	NONE	Q9BZL6
Uncharacterized protein HI1434	NONE	P45202
Outer membrane protein tolC	NONE	P02930
C-phycocyanin-1 alpha chain	NONE	P07122
Msx2-interacting protein	NONE	Q96T58
Homeobox protein Hox-B13	GENE REGULATION	HXB13_HUMA N
Growth factor receptor-bound protein 14	NONE	Q14449
G1/S-specific cyclin-E1	TRANSFERASE	CCNE1_HUMA N
CCR4-NOT transcription complex subunit 4	NONE	Q8BT14
Acyl-coenzyme A synthetase ACSM2A, mitochondrial	NONE	Q08AH3
Cullin-5	NONE	Q93034
4-alpha-glucanotransferase	NONE	O66937
Transcription factor E2F1	TUMOUR SUPPRESSOR	E2F1_HUMAN
Chloride intracellular channel protein 4	NONE	Q9Y696
Oligonucleotide/oligosaccharide-binding fold-containing protein 1	NONE	Q8K2X3
Zinc-alpha-2-glycoprotein	LIPID MOBILIZATION FACTOR	ZA2G_HUMA N
Carbamoyl-phosphate synthase large chain	NONE	P00968
Gigaxonin	NONE	Q9H2C0
RuvB-like 1	NONE	Q9Y265
Thiamine biosynthesis protein thiC	NONE	Q9A6Q5
E3 SUMO-protein ligase NSE2	NONE	Q96MF7

D-allulose-6-phosphate 3-epimerase	NONE	P32719
Exodeoxyribonuclease V gamma chain	NONE	P07648
D-lactate dehydrogenase	NONE	P30901
Fructose-bisphosphate aldolase B	ALDOLASE	ALDOB_HUMAN
DNA ligase	NONE	Q9ZHI0
Seed lipoxygenase-3	NONE	P09186
Retinol-binding protein 4	NONE	P18902
Hemagglutinin	NONE	P03452
Oxidoreductase	NONE	Q7SID9
Flavonol sulfotransferase-like	NONE	P52839
Uncharacterized protein yrbA	NONE	P0A9W6
Antitumor antibiotic C-1027 apoprotein	NONE	Q06110
Mitochondrial import inner membrane translocase subunit TIM44	NONE	Q01852
3-hydroxyisobutyryl-CoA hydrolase, mitochondrial	NONE	Q6NVY1
Endoglucanase C	NONE	P14090
Enoyl-CoA hydratase	NONE	Q5SLS5
Disks large homolog 1	NONE	Q62696
Pectate lyase	NONE	Q9X592
Ubiquitin-conjugating enzyme E2 variant 3	NONE	Q8IX04
Transaldolase	NONE	Q9WYD1
Penicillin G acylase	NONE	P06875
Protein S100-A6	SIGNALING PROTEIN	S10A6_HUMAN
Ubiquitin carboxyl-terminal hydrolase 19	HYDROLASE	UBP19_HUMAN
Zinc finger protein ZPR1	NONE	Q62384
Adenosine kinase	TRANSFERASE	ADK_HUMAN
Endoribonuclease Dicer	NONE	Q9UPY3
Pyrrolidone-carboxylate peptidase	NONE	O58321
Isocitrate lyase	NONE	P0A9G6

D-alanyl-D-alanine carboxypeptidase dacB	NONE	P24228
CUG-BP- and ETR-3-like factor 4	NONE	Q9BZC1
Endo-N-acetylneuraminidase	NONE	Q04830
Sulfite reductase, dissimilatory-type subunit alpha	NONE	P45574
Galectin-2	NONE	Q9P4R8
Exosome complex exonuclease RRP45	NONE	Q06265
V-type proton ATPase subunit H	NONE	P41807
tRNA (guanine-N(1)-)-methyltransferase	NONE	Q6G1R9
Nuclear inhibitor of protein phosphatase 1	NONE	Q8R3G1
Superoxide dismutase [Fe]	NONE	P18868
protein PA1000	NONE	P20581
Superoxide reductase	NONE	O58810
Fumarate reductase flavoprotein subunit	NONE	P17412
Inosine-5-monophosphate dehydrogenase	NONE	P50097
T-cell ecto-ADP-ribosyltransferase 2	NONE	P20974
Alpha-hemolysin translocation ATP-binding protein hlyB	NONE	P08716
2-oxoisovalerate dehydrogenase subunit alpha, mitochondrial	NONE	P12694
SLIT-ROBO Rho GTPase-activating protein 2	NONE	O75044
Platelet-activating factor acetylhydrolase IB subunit beta	HYDROLASE	PA1B2_HUMAN
Uncharacterized oxidoreductase ytbE	NONE	O34678
Feruloyl esterase A	NONE	O42807
mRNA-capping enzyme subunit alpha	NONE	P78587
DNA-directed RNA polymerases I, II, and III subunit RPABC3	NONE	P52434
NHP2-like protein 1	NONE	P55769
Rho guanine nucleotide exchange factor 12	SIGNALING PROTEIN/MEMBRANE PROTEIN	ARHGC_HUMAN
[Pyruvate dehydrogenase [lipoamide]] kinase isozyme 2, mitochondrial	TRANSFERASE	PDK2_HUMAN

Surface protein A	NONE	Q9RP17
Glycylpeptide N-tetradecanoyltransferase 1	NONE	P30419
Immunogenic protein MPT64	NONE	P0A5Q4
Helix-destabilizing protein	NONE	P03696
Vacuolar transporter chaperone 2	NONE	P43585
Arylamine N-acetyltransferase 1	TRANSFERASE	ARY1_HUMAN
Hydroxylamine reductase	NONE	Q01770
Choline O-acetyltransferase	NONE	P28329
Leucine-specific-binding protein	NONE	P04816
O-GlcNAcase BT_4395	NONE	Q89ZI2
Nitrilase and fragile histidine triad fusion protein NitFhit [Includes: Bis(5-adenosyl)-triphosphatase	NONE	O76463
Blue copper oxidase cueO	NONE	P36649
Protein kinase C theta type	TRANSFERASE	KPCT_HUMAN
Clavaminate synthase 1	NONE	Q05581
RING finger protein 24	NONE	Q9Y225

Sulfotransferase 1C2	TRANSFERASE	ST1C2_HUMAN
Alpha-amylase/trypsin inhibitor	NONE	P01087
Core protein VP7	NONE	P36325
60 kDa chaperonin 2	NONE	P0A520
Creatine kinase M-type	NONE	P04414
Peptide deformylase	NONE	Q9I7A8
3-dehydroquinate dehydratase	NONE	P15474
Alpha-amylase	NONE	P00692
ATP synthase subunit alpha, chloroplastic	NONE	P06450
Bifunctional protein birA synthetase	NONE	P06709
ATP-dependent molecular chaperone HSP82	NONE	P02829
Serine/threonine-protein phosphatase PP1-beta catalytic subunit	NONE	P62140
Ras-related protein Rab-27B	NONE	Q99P58
Probable diphthine synthase	NONE	O58456
Eukaryotic translation initiation factor 2 subunit 1	TRANSLATION	IF2A_HUMAN
Crotonobetainyl-CoA:carnitine CoA-transferase	NONE	P31572

Prostatic acid phosphatase	NONE	P20646
Cytochrome b6	NONE	P83791
Lysozyme C, milk isozyme	NONE	P11376
Manganese-dependent inorganic pyrophosphatase	NONE	Q58025
Vascular endothelial growth factor B	NONE	P49765
Fatty acid synthase subunit alpha	NONE	P19097
Baseplate protein	NONE	P08558
Aequorin-2	NONE	P02592
Heat-labile enterotoxin IIB, B chain	NONE	P43529
Sigma-E factor regulatory protein rseB	NONE	P0AFX9
NH(3)-dependent NAD(+) synthetase	NONE	Q3JL79
Delta-aminolevulinic acid dehydratase	NONE	P10518
1,3-beta-glucanosyltransferase GAS2	NONE	Q06135
Fructose-1,6-bisphosphatase 1	NONE	P00636
ADP-ribosylation factor-like protein 2-binding protein	NONE	Q7SYL1

Two-component system yycF/yycG regulatory protein yycH	NONE	Q794W0
Ribosomal RNA small subunit methyltransferase F	NONE	P76273
Transitional endoplasmic reticulum ATPase	NONE	Q01853
2,3,4,5-tetrahydropyridine-2,6-dicarboxylate N-succinyltransferase	NONE	Q8FV25
Ethanolamine utilization protein eutD	NONE	P77218
DNA polymerase beta	TRANSFERASE/DNA	DPOLB_HUMAN
Voltage-dependent L-type calcium channel subunit beta-4	NONE	O00305
Ubiquitin carboxyl-terminal hydrolase 14	NONE	Q8L6Y1
Serum albumin	TRANSPORT PROTEIN	ALBU_HUMAN
DNA primase small subunit	NONE	O57934
Nicotinamide riboside kinase 1	SIGNALING PROTEIN,TRANSFERASE	NRK1_HUMAN
Triosephosphate isomerase	NONE	P66940
Zinc finger and BTB domain-containing protein 43	NONE	O43298
Endoglucanase-5	NONE	P43316
Myosin-VI	NONE	Q29122

NEDD8 ultimate buster 1	NONE	P54729
Methionine gamma-lyase	NONE	P13254
Malate dehydrogenase	NONE	P80040
Ribulose bisphosphate carboxylase large chain	NONE	P00875
3-hydroxy-3-methylglutaryl CoA synthase	NONE	Q79ZY6
Putative 5(3)-deoxyribonucleotidase	NONE	Q8CTG7
Retinol-binding protein 3	NONE	Q7SZI7
o-succinylbenzoate synthase	NONE	P29208
Histone deacetylase-like amidohydrolase	NONE	Q70I53
GTP-binding protein era	NONE	P06616
Signal recognition particle 54 kDa protein	NONE	P14576
Glycyl-tRNA synthetase alpha subunit	NONE	Q9WY59
Sorting nexin-9	NONE	Q9Y5X1
Creatinase	NONE	P38488
Probable M18 family aminopeptidase 1	NONE	P0C925

Table S4: PharmMapper predicted protein targets for CID 5281643

Name	Class	Uniprot id
5-3 exoribonuclease 2	NONE	P40848
Cysteine protease avirulence protein avrPphB	NONE	Q52430
Peroxisome proliferator-activated receptor alpha	TRANSCRIPTION	PPARA_HUMAN
Myelin P2 protein	NONE	P0C6G6
Hemocyanin II	NONE	P04253
Ribonucleoside-diphosphate reductase small chain 1	NONE	P09938
Uncharacterized protein YKR043C	NONE	P36136
UPF0502 protein PSPTO_2686	NONE	Q882E2
3-isopropylmalate dehydratase small subunit	NONE	O59393
Endothelin-converting enzyme 2	NONE	O60344
DNA ligase	NONE	P00969
Transketolase	NONE	Q8MPM3
Protection of telomeres protein 1	NONE	Q9NUX5
Holliday junction ATP-dependent DNA helicase ruvB	NONE	Q56313
Aminotransferase ybdL	NONE	P77806
Acyl-CoA dehydrogenase family member 8, mitochondrial	NONE	Q9UKU7
1,3-beta-glucanosyltransferase GAS2	NONE	Q06135
Delta-aminolevulinic acid dehydratase	NONE	P0ACB2
Rho-related GTP-binding protein RhoQ	NONE	P17081
Uncharacterized lipoprotein gfcB	NONE	P75884
Interferon-induced guanylate-binding protein 1	NONE	P32455
Non-ATP-dependent L-selective hydantoinase	NONE	P81006
Hematopoietically-expressed homeobox protein HHEX	NONE	Q03014
Probable dipeptidyl-peptidase 3	NONE	Q08225
Vanadium haloperoxidase	NONE	P81701

Probable DNA primase small subunit	NONE	Q97Z83
G1/S-specific cyclin-D1	NONE	P24385
Cellular tumor antigen p53	NONE	P02340
Elongation factor Tu	NONE	P0A6N1
U4/U6 snRNA-associated-splicing factor PRP24	NONE	P49960
Serine/threonine-protein phosphatase PP1-beta catalytic subunit	NONE	P62140
Protein yibA	NONE	P0ADK7
Fumarylacetoacetase	NONE	P35505
Hemoglobin-binding protease hbp	NONE	O88093
Peptide-N(4)-(N-acetyl-beta-glucosaminyl)asparagine amidase	NONE	Q9JI78
Retinoic acid receptor beta	TRANSCRIPTION	RARB_HUMAN
Pyridoxal biosynthesis lyase pdxS	NONE	Q5SKD9
E3 ubiquitin-protein ligase RNF8	NONE	O76064
Fructose-1,6-bisphosphatase 1	NONE	P00636
D-alanyl-D-alanine carboxypeptidase dacB	NONE	P24228
Aspartic protease Bla g 2	NONE	P54958
Protein transport protein SEC23	NONE	P15303
Proto-oncogene vav	NONE	P27870
Protein translocase subunit secA	PROTEIN TRANSPORT	SECA_ECOLI
Arylphorin	NONE	Q7Z1F8
InaD-like protein	NONE	Q8NI35
Fatty acid synthase subunit alpha	NONE	P19097
Catalase	NONE	P77872
DNA (cytosine-5)-methyltransferase 1	NONE	P26358
L-asparaginase 1	NONE	P0A962
Oligonucleotide/oligosaccharide-binding fold-containing protein 1	NONE	Q8K2X3
Pyruvate, phosphate dikinase 1, chloroplastic	NONE	P11155
GAL10 bifunctional protein [Includes: UDP-glucose 4-epimerase	NONE	P04397

Beta-galactosidase	NONE	P00722
Heat shock protein homolog SSE1	NONE	P32589
Lanosterol synthase	ISOMERASE	ERG7_HUMAN
Penicillin G acylase	NONE	P06875
T-cell ecto-ADP-ribosyltransferase 2	NONE	P20974
Exocyst complex component 6	NONE	Q9VDE6
Putative acyl-CoA thioester hydrolase ybhC	NONE	P46130
Ras-related protein Rab-31	SIGNALING PROTEIN	RAB31_HUMAN
L-lysine 2,3-aminomutase	NONE	Q9XBQ8
Ribokinase	NONE	P0A9J6
Genome polyprotein	NONE	P12915
Transitional endoplasmic reticulum ATPase	NONE	Q01853
2-oxoisovalerate dehydrogenase subunit alpha, mitochondrial	NONE	P12694
Clavaminate synthase 1	NONE	Q05581
Outer membrane protein tolC	NONE	P02930
Zinc finger and BTB domain-containing protein 43	NONE	O43298
Probable fructose-2,6-bisphosphatase TIGAR B	NONE	Q7ZVE3
SUMO-conjugating enzyme UBC9	NONE	P63279
Citrate lyase beta subunit-like protein	NONE	O06162
Hypothetical protein yesE	NONE	O31511
4-alpha-glucanotransferase	NONE	O66937
Protein MJ0882	NONE	Q58292
Acrosin	NONE	P08001
StAR-related lipid transfer protein 5	NONE	Q9NSY2
HMG box-containing protein 1	NONE	O60381
Uracil phosphoribosyltransferase	NONE	O67914
Fibrinogen alpha-1 chain	NONE	P02674
G1/S-specific cyclin-E1	TRANSFERASE	CCNE1_HUMAN
52 kDa Ro protein	IMMUNOGLOBULIN DOMAIN	RO52_HUMAN
Uncharacterized oxidoreductase ytbE	NONE	O34678

Glucoamylase GLU1	NONE	P08017
L-2-hydroxyisocaproate dehydrogenase	NONE	P14295
UDP-N-acetylhexosamine pyrophosphorylase	NONE	Q16222
Peptide chain release factor 1	NONE	Q9X183
Gamma-glutamyl hydrolase	NONE	Q92820
Protein SEC13 homolog	NONE	P55735
L-lactate dehydrogenase C chain	NONE	P00342
Cold shock domain-containing protein E1	NONE	O75534
Antiviral protein 2	NONE	Q40772
Single-stranded DNA-binding protein	NONE	Q9WZ73
Sigma-E factor regulatory protein rseB	NONE	P0AFX9
Saccharopine dehydrogenase [NADP+, L-glutamate-forming]	NONE	Q9P4R4
tRNA-specific 2-thiouridylase mnmA	NONE	Q97T38
Exodeoxyribonuclease 8	NONE	P15032
Retinoid-binding protein 7	NONE	Q96R05
Myb-related protein B	NONE	Q03237
Glycyl-tRNA synthetase alpha subunit	NONE	Q9WY59
Glucosamine--fructose-6-phosphate aminotransferase [isomerizing] 1	NONE	Q06210
Glutamate [NMDA] receptor subunit 3A	NONE	Q9R1M7
Chloride intracellular channel exc-4	NONE	Q8WQA4
2,3,4,5-tetrahydropyridine-2,6-dicarboxylate N-succinyltransferase	NONE	Q8FV25
Arabinoxylan arabinofuranohydrolase	NONE	Q45071
E3 SUMO-protein ligase NSE2	NONE	Q96MF7
Trypanothione reductase	NONE	P39040
Phenoxazinone synthase	NONE	Q53692
Thiamine biosynthesis protein thiC	NONE	Q9A6Q5
Ribosome-inactivating protein alpha-trichosanthin	NONE	P09989
Docking protein 2	NONE	O60496

Cell division control protein 13	NONE	P32797
Mitogen-activated protein kinase kinase kinase 7-interacting protein 1	NONE	Q15750
Uncharacterized HTH-type transcriptional regulator yxaF	NONE	P42105
Ubiquitin carboxyl-terminal hydrolase 19	HYDROLASE	UBP19_HUMAN
FEZ-1 protein	NONE	Q9K578
Zinc finger protein ZPR1	NONE	Q62384
DNA/RNA-binding protein Alba 2	NONE	O28323
Primosomal replication protein n	NONE	P07013
Ribosomal RNA small subunit methyltransferase F	NONE	P76273
Xaa-Pro dipeptidyl-peptidase	NONE	P22346
Amidophosphoribosyltransferase	NONE	P00497
Homoserine O-acetyltransferase	NONE	P45131
Alcohol dehydrogenase, iron-containing	NONE	Q9X022
Elongation factor Tu-A	NONE	P60338
O-GlcNAcase BT_4395	NONE	Q89ZI2
Inositol-trisphosphate 3-kinase C	NONE	Q96DU7
NH(3)-dependent NAD(+) synthetase	NONE	O25096
Pancreatic lipase-related protein 1	NONE	P06857
Transcription factor SOX-5	NONE	P35710
Myosin-VI	NONE	Q29122
Pancreatic triacylglycerol lipase	NONE	P29183
NEDD8 ultimate buster 1	NONE	P54729
Endo-N-acetylneuraminidase	NONE	Q04830
Inosine-5-monophosphate dehydrogenase	NONE	P0C0H6
Spermidine synthase	NONE	P19623
Alpha-amylase type A isozyme	NONE	P00693
Putative ribosomal N-acetyltransferase ydaF	NONE	P96579
Sentrin-specific protease 7	HYDROLASE	SENP7_HUMAN
Guanine nucleotide-binding protein subunit alpha-13	NONE	P27601

Insulin-like growth factor 1 receptor	HORMONE RECEPTOR	IGF1R_HUMAN
Uncharacterized protein MJ1651	NONE	Q59045
Hexokinase	NONE	Q26609
Mitochondrial import inner membrane translocase subunit TIM21	NONE	P53220
Cyclin-dependent kinase 5 activator 1	COMPLEX(KINASE/ACTIVATOR)	CD5R1_HUMAN
CTP synthase	NONE	Q5SIA8
Glutamyl-tRNA(Gln) amidotransferase subunit A	NONE	P63488
Phosphoadenosine phosphosulfate reductase	NONE	O05927
Vascular endothelial growth factor A	COMPLEX (ANTIBODY/ANTIGEN)	VEGFA_HUMAN
Holliday junction ATP-dependent DNA helicase ruvA	NONE	Q9F1Q3
Alpha-amylase	NONE	P56634
Methyl-coenzyme M reductase I subunit alpha	NONE	Q49605
Zinc finger E-box-binding homeobox 2	NONE	O60315
[Pyruvate dehydrogenase [lipoamide]] kinase isozyme 2, mitochondrial	TRANSFERASE	PDK2_HUMAN
Nuclear cap-binding protein subunit 1	NONE	Q09161
Glucose-1-phosphate adenylyltransferase	NONE	P39669
L-arabinose isomerase	NONE	P08202
Tyrosine-protein kinase Lyn	NONE	P25911
Feruloyl esterase A	NONE	O42807
Neurogenic locus notch homolog protein 1	TRANSCRIPTION/DNA	NOTC1_HUMAN
Carboxypeptidase Y	NONE	P00729
Gibberellin receptor GID1	NONE	Q6L545
Glucose-6-phosphate isomerase	NONE	P06745
Chromosomal replication initiator protein dnaA	NONE	O66659

Spondin-1	NONE	Q9HCB6
3-dehydroquinate dehydratase	NONE	O66440
Methylthioribose-1-phosphate isomerase	NONE	Q06489
Poly [ADP-ribose] polymerase 1	NONE	Q9ZP54
Cytochrome c	NONE	P00142
ZZ-type zinc finger-containing protein 3	NONE	Q8IYH5
Ubiquitin thioesterase OTU1	NONE	P43558
Metallo-beta-lactamase L1	NONE	P52700
TNF receptor-associated factor 4	SIGNALING PROTEIN	TRAF4_HUMAN
DNA repair endonuclease XPF	DNA REPAIR, HYDROLASE	XPF_HUMAN
Tyrosine-protein kinase HCK	TRANSFERASE	HCK_HUMAN
Thermosome subunit alpha	NONE	P48424
Sulfite reductase, dissimilatory-type subunit alpha	NONE	P45574
Divalent-cation tolerance protein cutA	NONE	O28301
N-carbamoylsarcosine amidase	NONE	P32400

Dihydroxyacetone kinase	NONE	P45510
Lysozyme C, milk isozyme	NONE	P11376
Phosphoribosylamine--glycine ligase	NONE	O66949
Glucosamine--fructose-6-phosphate aminotransferase [isomerizing]	NONE	P53704
Isoleucyl-tRNA synthetase	LIGASE/RNA	SYI1_STAAU
Endoglucanase-5	NONE	P43316
Nucleolar transcription factor 1	NONE	P25976
3-hexulose-6-phosphate isomerase	NONE	P42404
Alpha-xylosidase	NONE	P31434
Signal recognition particle protein	NONE	O07347
L-ascorbate oxidase	NONE	P37064
DNA endonuclease I-CreI	NONE	P05725
Glutamate decarboxylase 2	NONE	Q05329
Chorismate synthase	NONE	P28777
Phospholipase A2 KPA2	NONE	Q9DF52

Splicing factor, arginine/serine-rich 1	NONE	Q07955
FACT complex subunit spt16	NONE	O94267
Cytochrome b6	NONE	P83791
Inositol-1,4,5-trisphosphate 5-phosphatase 1	NONE	O43001
Zinc finger protein 295	NONE	Q9ULJ3
3-hydroxyisobutyryl-CoA hydrolase, mitochondrial	NONE	Q6NVY1
23S rRNA (uracil-5-)-methyltransferase rumA	NONE	P55135
Amyloid beta A4 precursor protein-binding family B member 1	NONE	O00213
Nuclear migration protein nudC	NONE	O35685
Epsin-1	NONE	Q9Y6I3
Ribonuclease I	NONE	P21338
Ferredoxin--NADP reductase, chloroplastic	NONE	P00455
tRNA ribose 2-O-methyltransferase aTrm56	NONE	O58214
Polycomb protein Scm	NONE	Q9VHA0
L-aspartate oxidase	NONE	P10902

Malonyl CoA-acyl carrier protein transacylase	NONE	P63458
Methionine gamma-lyase	NONE	P13254
Homoserine kinase	NONE	Q58504
Aldo-keto reductase family 1 member C4	NONE	P17516
Ubiquitin-like protein 3	NONE	O95164
26S proteasome complex subunit DSS1	NONE	P60896
Endoglucanase E	NONE	P10477
Two-component system yycF/yycG regulatory protein yycH	NONE	Q794W0
Phospholipase A2 homolog zhaoermiatoxin	NONE	P84776
Sorbitol operon regulator	NONE	P37078
High mobility group protein B2	NONE	P17741
D-lactate dehydrogenase	OXIDOREDUCTASE	DLD_ECOLI
Protein MTH_152	NONE	O26255
Extracellular globin-4	NONE	P13579
Cyclic nucleotide-gated potassium channel mll3241	NONE	Q98GN8

Tryptophan synthase beta chain	NONE	P66984
SAM domain and HD domain-containing protein 1	NONE	Q9Y3Z3
Lactose permease	NONE	P02920
Filamin-B	NONE	O75369
Tyrosine-protein phosphatase non-receptor type 4	NONE	P29074
Botulinum neurotoxin type B	NONE	P10844
Phosphatase yidA	NONE	P0A8Y5
Ig mu chain C region	NONE	P01871
Glycogen phosphorylase, liver form	NONE	P06737
Zinc metalloproteinase/disintegrin	NONE	O57413
Ribonuclease HII	HYDROLASE	RNH2_METJA
Insulin-degrading enzyme	NONE	P14735
Heme-based aerotactic transducer hemAT	NONE	O07621
Exodeoxyribonuclease V gamma chain	NONE	P07648
Maltogenic amylase	NONE	O69007

Cytosol aminopeptidase	NONE	P68767
Lysozyme C	NONE	P00703
Riboflavin kinase	NONE	Q60365
UPF0310 protein PH1033	NONE	O58764
Oxygen-independent coproporphyrinogen-III oxidase	NONE	P32131
Alpha-hemolysin translocation ATP-binding protein hlyB	NONE	P08716
Uncharacterized protein yqeY	NONE	P54464
Phenylalanyl-tRNA synthetase alpha chain	NONE	Q4L5E3
72 kDa type IV collagenase	HYDROLASE	MMP2_HUMAN
Dual specificity protein phosphatase CDC14B	NONE	O60729
Cytochrome P450 2C5	NONE	P00179
Hereditary hemochromatosis protein	NONE	Q30201
Strictosidine synthase	NONE	P68175
Ribulose bisphosphate carboxylase large chain	NONE	P00877
Uridylate kinase	NONE	P59009

Beta-2-microglobulin	NONE	P61769
Peptidoglycan-recognition protein LC	NONE	Q9GNK5
ADP compounds hydrolase nudE	NONE	P45799
mRNA-capping enzyme subunit alpha	NONE	P78587
Malate dehydrogenase	NONE	P80039
Tail attachment protein	NONE	P03714
Bifunctional protein birA synthetase	NONE	P06709
UPF0244 protein VC_0702	NONE	Q9KU27
Hyaluronate lyase	NONE	Q53591
(S)-2-haloacid dehalogenase 4A	NONE	Q51645
Probable adenylyl-sulfate kinase	NONE	Q9YCR6
DNA polymerase I	NONE	P52026
Transcription factor tau 60 kDa subunit	NONE	Q12308
C-phycocyanin alpha chain	NONE	P00306
Zinc metalloproteinase aureolysin	NONE	P81177

Ferredoxin-dependent glutamate synthase 2	NONE	P55038
Histone deacetylase-like amidohydrolase	NONE	Q70I53
Protein-L-isoaspartate O-methyltransferase	NONE	Q56308
Methylmalonyl-CoA carboxyltransferase 12S subunit	NONE	Q8GBW6
Conserved oligomeric Golgi complex subunit 2	NONE	P53271
Uncharacterized protein ydfO	NONE	P76156
Aspartate aminotransferase, cytoplasmic	NONE	P00504
Aminoacyltransferase femA	NONE	P0A0A5
2,3-bisphosphoglycerate-independent phosphoglycerate mutase	NONE	Q81X77
Adenylate cyclase	NONE	P94182
Ras-related C3 botulinum toxin substrate 1	SIGNALING PROTEIN,APOPTOSIS/HYDROLASE	RAC1_HUMAN
Dipeptidyl peptidase 4	HYDROLASE	DPP4_HUMAN
30S ribosomal protein S24e	NONE	Q8PZ95
Fatty acid/phospholipid synthesis protein plsX	NONE	P71018
Contactin-2	NONE	Q02246

Citrate synthase	NONE	Q53554
Methanol dehydrogenase subunit 1	NONE	P16027
Guanine nucleotide exchange factor VAV3	NONE	Q9UKW4
Serine hydroxymethyltransferase 1	NONE	O53441
Inositol-tetrakisphosphate 1-kinase	NONE	Q13572
Extracellular giant hemoglobin major globin subunit A1	NONE	Q7M419
Alpha-amylase A type-1/2	NONE	P0C1B3
Aequorin-2	NONE	P02592
Glyceraldehyde-3-phosphate dehydrogenase 1	NONE	P84998
Endo-1,4-beta-xylanase A	NONE	P18429
Trimethylamine dehydrogenase	NONE	P16099
Globin C, coelomic	NONE	P80018
Protein SMG7	NONE	Q92540
UV excision repair protein RAD23 homolog B	NONE	P54727

Table S5: Filtered predicted bacterial 80 protein targets for CID 72277

Entry	Protein names
Q70I53	Histone deacetylase-like amidohydrolase (HDAC-like amidohydrolase) (HDAH) (EC 3.5.1.-)
P00692	Alpha-amylase (EC 3.2.1.1) (1,4-alpha-D-glucan glucanohydrolase)
Q81JJ9	GMP reductase (EC 1.7.1.7) (Guanosine 5'-monophosphate oxidoreductase) (Guanosine monophosphate reductase)
O31641	Uncharacterized protein YjcS
O31698	Cyclic di-AMP receptor B (c-di-AMP receptor B) (c-di-AMP receptor protein DarB)
O31858	Uncharacterized protein YojF
O34533	Uncharacterized HTH-type transcriptional regulator YtcD
O34678	Uncharacterized oxidoreductase YtbE (EC 1.-.-.-)
P28366	Protein translocase subunit SecA (EC 7.4.2.8)
P96579	Putative ribosomal N-acetyltransferase YdaF (EC 2.3.1.-)
Q45071	Arabinoxylan arabinofuranohydrolase (AXH) (EC 3.2.1.55) (AXH-m2,3) (AXH-m23) (Alpha-L-arabinofuranosidase) (AF)
Q794W0	Two-component system WalR/WalK regulatory protein YycH
Q8FV25	2,3,4,5-tetrahydropyridine-2,6-dicarboxylate N-succinyltransferase (EC 2.3.1.117) (Tetrahydrodipicolinate N-succinyltransferase) (THDP succinyltransferase) (THP succinyltransferase) (Tetrahydropicolinate succinylase)
P06875	Penicillin G acylase (EC 3.5.1.11) (Penicillin G amidase) (Penicillin G amidohydrolase) [Cleaved into: Penicillin G acylase subunit alpha; Penicillin G acylase subunit beta]
P08716	Alpha-hemolysin translocation ATP-binding protein HlyB
P13988	Transposon Tn7 transposition protein TnsA (Restriction enzyme-like endonuclease TnsA) (EC 3.1.21.-)
P43529	Heat-labile enterotoxin IIB, B chain (LT-IIB)
P00968	Carbamoyl phosphate synthase large chain (EC 6.3.4.16) (EC 6.3.5.5) (Carbamoyl phosphate synthetase ammonia chain)
P02930	Outer membrane protein TolC (Multidrug efflux pump subunit TolC) (Outer membrane factor TolC)
P04816	Leucine-specific-binding protein (L-BP) (LS-BP)
P06616	GTPase Era (ERA) (GTP-binding protein Era)
P06709	Bifunctional ligase/repressor BirA (Biotin operon repressor) (Biotin--[acetyl-CoA-carboxylase] ligase) (EC 6.3.4.15) (Biotin--protein ligase) (Biotin-[acetyl-CoA carboxylase] synthetase)
P06986	Histidinol-phosphate aminotransferase (EC 2.6.1.9) (Imidazole acetol-phosphate transaminase) (HPAT) (HspAT)
P07648	RecBCD enzyme subunit RecC (Exodeoxyribonuclease V 125 kDa polypeptide) (Exodeoxyribonuclease V gamma chain) (Exonuclease V subunit RecC) (ExoV subunit RecC) (Helicase/nuclease RecBCD subunit RecC)
P0A9G6	Isocitrate lyase (ICL) (EC 4.1.3.1) (Isocitrase) (Isocitratase)

P0A9W6	Acid stress protein IbaG
P0ABQ0	Coenzyme A biosynthesis bifunctional protein CoaBC (DNA/pantothenate metabolism flavoprotein) (Phosphopantothenoylcysteine synthetase/decarboxylase) (PPCS-PPCDC) [Includes: Phosphopantothenoylcysteine decarboxylase (PPC decarboxylase) (PPC-DC) (EC 4.1.1.36) (CoaC); Phosphopantothenate--cysteine ligase (EC 6.3.2.5) (CoaB) (Phosphopantothenoylcysteine synthetase) (PPC synthetase) (PPC-S)]
P0AFX9	Sigma-E factor regulatory protein RseB
P0AGC3	Soluble lytic murein transglycosylase (EC 4.2.2.n1) (Exomuramidase) (Peptidoglycan lytic exotransglycosylase) (Slt70)
P0C0S1	Small-conductance mechanosensitive channel
P15032	Exodeoxyribonuclease 8 (EC 3.1.11.-) (Exodeoxyribonuclease VIII) (EXO VIII)
P24228	D-alanyl-D-alanine carboxypeptidase DacB (DD-carboxypeptidase) (DD-peptidase) (EC 3.4.16.4) (D-alanyl-D-alanine endopeptidase) (DD-endopeptidase) (EC 3.4.21.-) (Penicillin-binding protein 4) (PBP-4)
P29208	o-succinylbenzoate synthase (OSB synthase) (OSBS) (EC 4.2.1.113) (4-(2'-carboxyphenyl)-4-oxybutyric acid synthase) (o-succinylbenzoic acid synthase)
P31572	L-carnitine CoA-transferase (EC 2.8.3.21) (Crotonobetainyl-CoA:carnitine CoA-transferase)
P32719	D-allulose-6-phosphate 3-epimerase (EC 5.1.3.-)
P36649	Multicopper oxidase CueO (MCO) (EC 1.16.3.4) (Blue copper oxidase CueO) (Copper efflux oxidase) (Cu efflux oxidase) (Cuprous oxidase)
P39377	Isoaspartyl dipeptidase (EC 3.4.19.-)
P46130	Putative acyl-CoA thioester hydrolase YbhC (EC 3.1.2.-)
P76273	Ribosomal RNA small subunit methyltransferase F (EC 2.1.1.178) (16S rRNA m5C1407 methyltransferase) (rRNA (cytosine-C(5)-)-methyltransferase RsmF)
P76537	Uncharacterized protein YfeY
P76562	tRNA(Met) cytidine acetyltransferase TmcA (EC 2.3.1.193) (Lysine 2-hydroxyisobutyryltransferase) (EC 2.3.1.-)
P77218	Phosphate acetyltransferase EutD (EC 2.3.1.8) (Ethanolamine utilization protein EutD)
Q04830	Tail spike protein (TSP) (Endo-N-acetylneuraminidase) (Endo-N) (Endo-alpha-sialidase) (EC 3.2.1.129) (EndoNF) (G102) [Cleaved into: Mature tail spike protein; Intramolecular chaperone]
P08558	Baseplate hub protein gp44 (43 kDa tail protein) (Gene product 44) (gp44) (Gene product P) (gpP)
P03696	Single-stranded DNA-binding protein (SSB protein) (2.5 protein) (Gene product 2.5) (gp2.5)
Q5KY26	Glutaminase (EC 3.5.1.2)
O31410	Peptide deformylase 2 (PDF 2) (EC 3.5.1.88) (Polypeptide deformylase 2)
P45083	Putative esterase HI_1161 (EC 3.1.2.-)
P45202	Cys-tRNA(Pro)/Cys-tRNA(Cys) deacylase YbaK (EC 4.2.-.-)
P30901	D-lactate dehydrogenase (D-LDH) (EC 1.1.1.28) (D-specific 2-hydroxyacid dehydrogenase)
P22346	Xaa-Pro dipeptidyl-peptidase (EC 3.4.14.11) (X-Pro dipeptidyl-peptidase) (X-prolyl-dipeptidyl aminopeptidase) (X-PDAP)
P11411	Glucose-6-phosphate 1-dehydrogenase (G6PD) (EC 1.1.1.363) (Glucose-6-phosphate dehydrogenase (NAD(P)(+)))

P9WG42	Triosephosphate isomerase (TIM) (TPI) (EC 5.3.1.1) (Triose-phosphate isomerase)
P9WG43	Triosephosphate isomerase (TIM) (TPI) (EC 5.3.1.1) (Triose-phosphate isomerase)
P9WGI8	Serine hydroxymethyltransferase 1 (SHM1) (SHMT 1) (Serine methylase 1) (EC 2.1.2.1)
P9WGI9	Serine hydroxymethyltransferase 1 (SHM1) (SHMT 1) (Serine methylase 1) (EC 2.1.2.1)
P9WGZ6	Ribonuclease PH (RNase PH) (EC 2.7.7.56) (tRNA nucleotidyltransferase)
P9WGZ7	Ribonuclease PH (RNase PH) (EC 2.7.7.56) (tRNA nucleotidyltransferase)
P9WIN8	Immunogenic protein MPT64 (Antigen MPT64)
P9WIN9	Immunogenic protein MPT64 (Antigen MPT64)
P9WPE6	Chaperonin GroEL 2 (EC 5.6.1.7) (60 kDa chaperonin 2) (65 kDa antigen) (Antigen A) (Cell wall protein A) (Chaperonin-60 2) (Cpn60 2) (Heat shock protein 65)
P9WPE7	Chaperonin GroEL 2 (EC 5.6.1.7) (60 kDa chaperonin 2) (65 kDa antigen) (Antigen A) (Cell wall protein A) (Chaperonin-60 2) (Cpn60 2) (Heat shock protein 65) (HSP65) [Cleaved into: Cleaved form]
P0C558	DNA protection during starvation protein (EC 1.16.-.-)
Q9RP17	Surface protein A
P20581	2-aminobenzoylacetyl-CoA thioesterase (EC 3.1.2.32)
Q9I7A8	Peptide deformylase (PDF) (EC 3.5.1.88) (Polypeptide deformylase)
P13254	L-methionine gamma-lyase (MGL) (EC 4.4.1.11) (Homocysteine desulfhydrase) (EC 4.4.1.2) (L-methioninase)
P38488	Creatinase (EC 3.5.3.3) (Creatine amidinohydrolase)
Q52430	Cysteine protease avirulence protein AvrPphB (EC 3.4.22.-) [Cleaved into: 7 kDa product; 28 kDa product]
P0A079	Methionine aminopeptidase (MAP) (MetAP) (EC 3.4.11.18) (Peptidase M)
Q6GFF9	Diacylglycerol kinase (DAG kinase) (DAGK) (EC 2.7.1.107)
Q8CTG7	Putative 5'(3')-deoxyribonucleotidase (EC 3.1.3.-)
Q4L5E3	Phenylalanine--tRNA ligase alpha subunit (EC 6.1.1.20) (Phenylalanyl-tRNA synthetase alpha subunit) (PheRS)
P95780	dTDP-glucose 4,6-dehydratase (EC 4.2.1.46)
Q8CWX6	5-methyltetrahydropteroyltriglutamate--homocysteine methyltransferase (EC 2.1.1.14) (Cobalamin-independent methionine synthase) (Methionine synthase, vitamin-B12 independent isozyme)
Q54873	Hyaluronate lyase (EC 4.2.2.1) (Hyaluronidase) (HYase)
Q05581	Clavaminate synthase 1 (EC 1.14.11.21) (Clavaminic acid synthase 1) (CAS1) (CS1)
P15474	3-dehydroquinate dehydratase (3-dehydroquinase) (EC 4.2.1.10) (Type II DHQase)
Q06110	Antitumor antibiotic C-1027 apoprotein (C-1027-AG)
P14295	L-2-hydroxyisocaproate dehydrogenase (L-HicDH) (EC 1.1.1.-)

Table S6: Filtered predicted bacterial 77 protein targets for CID 5281643

Entry	Protein names	Organism (ID)
Q81X77	2,3-bisphosphoglycerate-independent phosphoglycerate mutase (BPG-independent PGAM) (Phosphoglyceromutase) (iPGM) (EC 5.4.2.12)	Bacillus anthracis
O31511	Uncharacterized protein YesE	Bacillus subtilis (strain 168)
O34678	Uncharacterized oxidoreductase YtbE (EC 1.-.-.-)	Bacillus subtilis (strain 168)
Q45071	Arabinoxylan arabinofuranohydrolase (AXH) (EC 3.2.1.55) (AXH-m2,3) (AXH-m23) (Alpha-L-arabinofuranosidase) (AF)	Bacillus subtilis (strain 168)
P42105	Uncharacterized HTH-type transcriptional regulator YxaF	Bacillus subtilis (strain 168)
P00497	Amidophosphoribosyltransferase (ATase) (EC 2.4.2.14) (Glutamine phosphoribosylpyrophosphate amidotransferase) (GPATase)	Bacillus subtilis (strain 168)
P96579	Putative ribosomal N-acetyltransferase YdaF (EC 2.3.1.-)	Bacillus subtilis (strain 168)
P42404	3-hexulose-6-phosphate isomerase (EC 5.3.1.27) (6-phospho-3-hexuloisomerase) (PHI)	Bacillus subtilis (strain 168)
Q794W0	Two-component system WalR/WalK regulatory protein YycH	Bacillus subtilis (strain 168)
O07621	Heme-based aerotactic transducer HemAT	Bacillus subtilis (strain 168)
P54464	Uncharacterized protein YqeY	Bacillus subtilis (strain 168)
P71018	Phosphate acyltransferase (EC 2.3.1.274) (Acyl-ACP phosphotransacylase) (Acyl-[acyl-carrier-protein]--phosphate acyltransferase) (Phosphate-acyl-ACP acyltransferase)	Bacillus subtilis (strain 168)
P18429	Endo-1,4-beta-xylanase A (Xylanase A) (EC 3.2.1.8) (1,4-beta-D-xylan xylanohydrolase A)	Bacillus subtilis (strain 168)
Q8FV25	2,3,4,5-tetrahydropyridine-2,6-dicarboxylate N-succinyltransferase (EC 2.3.1.117) (Tetrahydrodipicolinate N-succinyltransferase) (THDP succinyltransferase) (THP succinyltransferase) (Tetrahydropicolinate succinylase)	Brucella suis biovar 1 (strain 1330)
Q51645	(S)-2-haloacid dehalogenase 4A (EC 3.8.1.2) (2-haloalkanoic acid dehalogenase IVA) (Halocarboxylic acid halidohydrolase IVA) (L-2-haloacid dehalogenase IVA)	Burkholderia cepacia (Pseudomonas cepacia)
P10844	Botulinum neurotoxin type B (BoNT/B) (Bontoxilysin-B) [Cleaved into: Botulinum neurotoxin B light chain (LC) (EC 3.4.24.69); Botulinum neurotoxin B heavy chain (HC)]	Clostridium botulinum
Q9XBQ8	L-lysine 2,3-aminomutase (LAM) (EC 5.4.3.2) (KAM)	Clostridium subterminale
O88093	Hemoglobin-binding protease hbp autotransporter (EC 3.4.21.-) [Cleaved into: Hemoglobin-binding protease hbp; Hemoglobin-binding protease hbp translocator (Helper peptide)]	Escherichia coli
P06875	Penicillin G acylase (EC 3.5.1.11) (Penicillin G amidase) (Penicillin G amidohydrolase) [Cleaved into: Penicillin G acylase subunit alpha; Penicillin G acylase subunit beta]	Escherichia coli
P08716	Alpha-hemolysin translocation ATP-binding protein HlyB	Escherichia coli
P77806	Methionine aminotransferase (EC 2.6.1.88) (Methionine-oxo-acid transaminase)	Escherichia coli (strain K12)

P0ACB2	Delta-aminolevulinic acid dehydratase (ALAD) (ALADH) (EC 4.2.1.24) (Porphobilinogen synthase)	Escherichia coli (strain K12)
P75884	Uncharacterized lipoprotein GfcB (Group 4 capsule protein B homolog)	Escherichia coli (strain K12)
P0CE48	Elongation factor Tu 2 (EF-Tu 2) (EC 3.6.5.3) (Bacteriophage Q beta RNA-directed RNA polymerase subunit III) (P-43)	Escherichia coli (strain K12)
P0CE47	Elongation factor Tu 1 (EF-Tu 1) (EC 3.6.5.3) (Bacteriophage Q beta RNA-directed RNA polymerase subunit III) (P-43)	Escherichia coli (strain K12)
P24228	D-alanyl-D-alanine carboxypeptidase DacB (DD-carboxypeptidase) (DD-peptidase) (EC 3.4.16.4) (D-alanyl-D-alanine endopeptidase) (DD-endopeptidase) (EC 3.4.21.-) (Penicillin-binding protein 4) (PBP-4)	Escherichia coli (strain K12)
P10408	Protein translocase subunit SecA (EC 7.4.2.8)	Escherichia coli (strain K12)
P0A962	L-asparaginase 1 (EC 3.5.1.1) (L-asparaginase I) (L-ASNase I) (L-asparagine amidohydrolase I)	Escherichia coli (strain K12)
P00722	Beta-galactosidase (Beta-gal) (EC 3.2.1.23) (Lactase)	Escherichia coli (strain K12)
P46130	Putative acyl-CoA thioester hydrolase YbhC (EC 3.1.2.-)	Escherichia coli (strain K12)
P0A9J6	Ribokinase (RK) (EC 2.7.1.15)	Escherichia coli (strain K12)
P02930	Outer membrane protein TolC (Multidrug efflux pump subunit TolC) (Outer membrane factor TolC)	Escherichia coli (strain K12)
P0AFX9	Sigma-E factor regulatory protein RseB	Escherichia coli (strain K12)
P15032	Exodeoxyribonuclease 8 (EC 3.1.11.-) (Exodeoxyribonuclease VIII) (EXO VIII)	Escherichia coli (strain K12)
P07013	Primosome protein PriB (Primosomal replication protein n)	Escherichia coli (strain K12)
P76273	Ribosomal RNA small subunit methyltransferase F (EC 2.1.1.178) (16S rRNA m5C1407 methyltransferase) (rRNA (cytosine-C(5)-)-methyltransferase RsmF)	Escherichia coli (strain K12)
P08202	L-arabinose isomerase (EC 5.3.1.4)	Escherichia coli (strain K12)
P31434	Alpha-xylosidase (EC 3.2.1.177)	Escherichia coli (strain K12)
P55135	23S rRNA (uracil(1939)-C(5))-methyltransferase RlmD (EC 2.1.1.190) (23S rRNA(m5U1939)-methyltransferase)	Escherichia coli (strain K12)
P21338	Ribonuclease I (RNase I) (EC 4.6.1.21) (Enterobacter ribonuclease)	Escherichia coli (strain K12)
P10902	L-aspartate oxidase (LASPO) (EC 1.4.3.16) (L-aspartate:fumarate oxidoreductase) (EC 1.5.99.-) (Quinolinate synthetase B)	Escherichia coli (strain K12)
P06149	Quinone-dependent D-lactate dehydrogenase (EC 1.1.5.12) ((R)-lactate:quinone 2-oxidoreductase) (D-lactate dehydrogenase) (D-LDH) (Respiratory D-lactate dehydrogenase)	Escherichia coli (strain K12)
P02920	Lactose permease (Lactose-proton symport)	Escherichia coli (strain K12)
P0A8Y5	Sugar phosphatase YidA (EC 3.1.3.23)	Escherichia coli (strain K12)

P07648	RecBCD enzyme subunit RecC (Exodeoxyribonuclease V 125 kDa polypeptide) (Exodeoxyribonuclease V gamma chain) (Exonuclease V subunit RecC) (ExoV subunit RecC) (Helicase/nuclease RecBCD subunit RecC)	Escherichia coli (strain K12)
P68767	Cytosol aminopeptidase (EC 3.4.11.1) (Aminopeptidase A/I) (Leucine aminopeptidase) (LAP) (EC 3.4.11.10) (Leucyl aminopeptidase)	Escherichia coli (strain K12)
P32131	Oxygen-independent coproporphyrinogen III oxidase (CPO) (EC 1.3.98.3) (Coproporphyrinogen III dehydrogenase) (CPDH)	Escherichia coli (strain K12)
P45799	ADP compounds hydrolase NudE (EC 3.6.1.-)	Escherichia coli (strain K12)
P06709	Bifunctional ligase/repressor BirA (Biotin operon repressor) (Biotin--[acetyl-CoA-carboxylase] ligase) (EC 6.3.4.15) (Biotin--protein ligase) (Biotin-[acetyl-CoA carboxylase] synthetase)	Escherichia coli (strain K12)
P76156	Uncharacterized protein YdfO	Escherichia coli (strain K12)
P0ADK7	Protein YibA	Escherichia coli O157:H7
P52026	DNA polymerase I (POL I) (EC 2.7.7.7)	Geobacillus stearothermophilus (Bacillus stearothermophilus)
P45131	Homoserine O-acetyltransferase (HAT) (EC 2.3.1.31) (Homoserine O-trans-acetylase) (Homoserine transacetylase) (HTA)	Haemophilus influenzae (strain ATCC 51907 / DSM 11121 / KW20 / Rd)
P77872	Catalase (EC 1.11.1.6)	Helicobacter pylori (strain ATCC 700392 / 26695) (Campylobacter pylori)
O25096	NH(3)-dependent NAD(+) synthetase (EC 6.3.1.5)	Helicobacter pylori (strain ATCC 700392 / 26695) (Campylobacter pylori)
P9WPE1	Citrate lyase subunit beta-like protein (EC 4.1.-.-)	Mycobacterium tuberculosis (strain ATCC 25618 / H37Rv)
P9WNG5	Malonyl CoA-acyl carrier protein transacylase (MCT) (EC 2.3.1.39)	Mycobacterium tuberculosis (strain ATCC 25618 / H37Rv)
P9WFX9	Tryptophan synthase beta chain (EC 4.2.1.20)	Mycobacterium tuberculosis (strain ATCC 25618 / H37Rv)
P9WGI9	Serine hydroxymethyltransferase 1 (SHM1) (SHMT 1) (Serine methylase 1) (EC 2.1.2.1)	Mycobacterium tuberculosis (strain ATCC 25618 / H37Rv)
P9WPE0	Citrate lyase subunit beta-like protein (EC 4.1.-.-)	Mycobacterium tuberculosis (strain CDC 1551 / Oshkosh)

P9WNG4	Malonyl CoA-acyl carrier protein transacylase (MCT) (EC 2.3.1.39)	Mycobacterium tuberculosis (strain CDC 1551 / Oshkosh)
P9WFX8	Tryptophan synthase beta chain (EC 4.2.1.20)	Mycobacterium tuberculosis (strain CDC 1551 / Oshkosh)
P9WGI8	Serine hydroxymethyltransferase 1 (SHM1) (SHMT 1) (Serine methylase 1) (EC 2.1.2.1)	Mycobacterium tuberculosis (strain CDC 1551 / Oshkosh)
O05927	Adenosine 5'-phosphosulfate reductase (APS reductase) (EC 1.8.4.10) (5'-adenylylsulfate reductase) (Thioredoxin-dependent 5'-adenylylsulfate reductase)	Pseudomonas aeruginosa (strain ATCC 15692 / DSM 22644 / CIP 104116 / JCM 14847 / LMG 12228 / 1C / PRS 101 / PAO1)
P13254	L-methionine gamma-lyase (MGL) (EC 4.4.1.11) (Homocysteine desulfhydrase) (EC 4.4.1.2) (L-methioninase)	Pseudomonas putida (Arthrobacter siderocapsulatus)
Q52430	Cysteine protease avirulence protein AvrPphB (EC 3.4.22.-) [Cleaved into: 7 kDa product; 28 kDa product]	Pseudomonas savastanoi pv. phaseolicola (Pseudomonas syringae pv. phaseolicola)
Q882E2	UPF0502 protein PSPTO_2686	Pseudomonas syringae pv. tomato (strain ATCC BAA-871 / DC3000)
P41972	Isoleucine--tRNA ligase (EC 6.1.1.5) (Isoleucyl-tRNA synthetase) (IleRS)	Staphylococcus aureus
P81177	Zinc metalloproteinase aureolysin (EC 3.4.24.29) (Staphylococcus aureus neutral proteinase)	Staphylococcus aureus
P0A0A5	Aminoacyltransferase FemA (EC 2.3.2.17) (Factor essential for expression of methicillin resistance A) (N-acetylmuramoyl-L-alanyl-D-glutamyl-L-lysyl-(N6-glycyl)-D-alanyl-D-alanine-diphosphoundecaprenyl-N-acetylglucosamine:glycine glycyltransferase)	Staphylococcus aureus
P63488	Glutamyl-tRNA(Gln) amidotransferase subunit A (Glu-ADT subunit A) (EC 6.3.5.7)	Staphylococcus aureus (strain Mu50 / ATCC 700699)
Q4L5E3	Phenylalanine--tRNA ligase alpha subunit (EC 6.1.1.20) (Phenylalanyl-tRNA synthetase alpha subunit) (PheRS)	Staphylococcus haemolyticus (strain JCSC1435)
Q53591	Hyaluronate lyase (EC 4.2.2.1) (Hyaluronidase) (HYase)	Streptococcus agalactiae serotype III (strain NEM316)
Q97T38	tRNA-specific 2-thiouridylase MnmA (EC 2.8.1.13)	Streptococcus pneumoniae serotype 4 (strain ATCC BAA-334 / TIGR4)
Q53692	O-aminophenol oxidase (EC 1.10.3.4) (Phenoxazinone synthase) (PHS)	Streptomyces antibioticus
Q05581	Clavaminate synthase 1 (EC 1.14.11.21) (Clavaminic acid synthase 1) (CAS1) (CS1)	Streptomyces clavuligerus

Q9KU27	Inosine/xanthosine triphosphatase (ITPase/XTPase) (EC 3.6.1.73) (Non-canonical purine NTP phosphatase) (Non-standard purine NTP phosphatase) (Nucleoside-triphosphate phosphatase) (NTPase)	Vibrio cholerae serotype O1 (strain ATCC 39315 / El Tor Inaba N16961)
